# Distinct and Specific Role of NlpC/P60 Endopeptidases LytA and LytB in Cell Elongation and Division of *Lactobacillus plantarum*

**DOI:** 10.3389/fmicb.2019.00713

**Published:** 2019-04-12

**Authors:** Marie-Clémence Duchêne, Thomas Rolain, Adrien Knoops, Pascal Courtin, Marie-Pierre Chapot-Chartier, Yves F. Dufrêne, Bernard F. Hallet, Pascal Hols

**Affiliations:** ^1^Louvain Institute of Biomolecular Science and Technology, Université Catholique de Louvain, Louvain-La-Neuve, Belgium; ^2^Micalis Institute, INRA, AgroParisTech, Université Paris-Saclay, Jouy-en-Josas, France

**Keywords:** *Lactobacillus*, cell cycle, cell wall, muropeptidase, NlpC/P60 endopeptidase, peptidoglycan hydrolase, morphogenesis

## Abstract

Peptidoglycan (PG) is an essential lattice of the bacterial cell wall that needs to be continuously remodeled to allow growth. This task is ensured by the concerted action of PG synthases that insert new material in the pre-existing structure and PG hydrolases (PGHs) that cleave the PG meshwork at critical sites for its processing. Contrasting with *Bacillus subtilis* that contains more than 35 PGHs, *Lactobacillus plantarum* is a non-sporulating rod-shaped bacterium that is predicted to possess a minimal set of 12 PGHs. Their role in morphogenesis and cell cycle remains mostly unexplored, except for the involvement of the glucosaminidase Acm2 in cell separation and the NlpC/P60 D, L-endopeptidase LytA in cell shape maintenance. Besides LytA, *L. plantarum* encodes three additional NlpC/P60 endopeptidases (i.e., LytB, LytC and LytD). The *in silico* analysis of these four endopeptidases suggests that they could have redundant functions based on their modular organization, forming two pairs of paralogous enzymes. In this work, we investigate the role of each Lyt endopeptidase in cell morphogenesis in order to evaluate their distinct or redundant functions, and eventually their synthetic lethality. We show that the paralogous LytC and LytD enzymes are not required for cell shape maintenance, which may indicate an accessory role such as in PG recycling. In contrast, LytA and LytB appear to be key players of the cell cycle. We show here that LytA is required for cell elongation while LytB is involved in the spatio-temporal regulation of cell division. In addition, both PGHs are involved in the proper positioning of the division site. The absence of LytA activity is responsible for the asymmetrical positioning of septa in round cells while the lack of LytB results in a lateral misplacement of division planes in rod-shaped cells. Finally, we show that the co-inactivation of LytA and LytB is synthetically affecting cell growth, which confirms the key roles played by both enzymes in PG remodeling during the cell cycle of *L. plantarum*. Based on the large distribution of NlpC/P60 endopeptidases in low-GC Gram-positive bacteria, these enzymes are attractive targets for the discovery of novel antimicrobial compounds.

## Introduction

The cell wall is a rigid structure that protects bacteria against external and internal pressures while giving them a proper shape (Delcour et al., 1999). A major component of the cell wall is the peptidoglycan (PG). The PG is a polymer composed of glycan strands linked together by peptide side chains to form a meshwork. The composition of the glycan strands is the same in all bacterial species: *N*-acetyl-muramic acid (MurNAc) alternating with *N*-acetyl-glucosamine (GlcNAc), which are connected by β-1,4 linkage (Vollmer, 2008). The composition of the peptide side chain is more flexible and can vary between bacterial species (Vollmer et al., 2008a). In *Lactobacillus plantarum*, the composition of the stem peptide is L-Ala, D-Glu, *meso*-diaminopimelate (*meso*-DAP), D-Ala, and D-lactate (Deghorain et al., 2007; Kleerebezem et al., 2010). The PG of *L. plantarum* is decorated with additional elements such as wall teichoic acids (WTA), *O*-acetylation of MurNAc (39%) and GlcNAc (9%) (Bernard et al., 2011a), and amidation of D-Glu (100%) and *meso*-DAP (94%) (Bernard et al., 2011b).

Bacteria have to continuously adapt the composition and organization of the PG in order to grow and divide properly. This task is performed by the concerted action between PG synthases and PG hydrolases (PGHs). While PG synthases mediate two main enzymatic reactions, i.e., transglycosylation and transpeptidation, PGHs have a broad range of enzymatic activities with respect to their role in PG processing (Vollmer et al., 2008b). They are separated into different families according to their PG cleavage sites (Vollmer et al., 2008b). Three families of enzymes cleave the glycan strand: glucosaminidases hydrolyze the sugar bond between GlcNAc and MurNAc (Litzinger et al., 2010); muramidases and lytic transglycosylases cleave between MurNAc and GlcNAc (Höltje et al., 1975; Barrett et al., 1984). Amidases cleave the amide bond between the glycan strand and the peptide side chain (Vollmer et al., 2008b). The last family is composed of endopeptidases (Vollmer et al., 2008b) and carboxypeptidases (Sauvage et al., 2008) that hydrolyze specific links in the peptide side chain or the interpeptide bridge.

**Figure 1:**
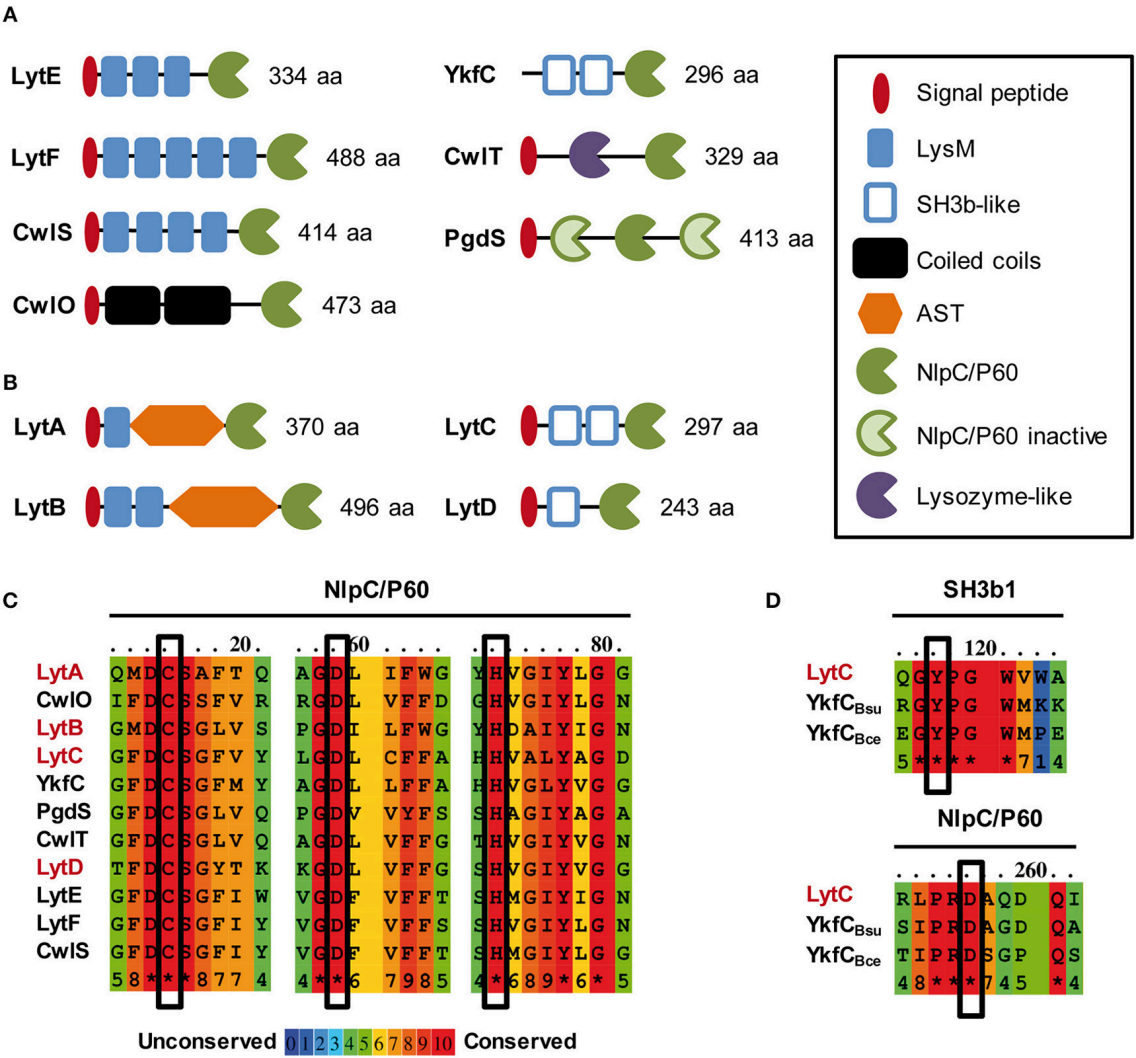
*In silico* analysis of NlpC/P60 endopeptidases of *B. subtilis* and *L. plantarum*. Schematic representation of LytE, LytF, CwlS, CwlO, CwlT and PgdS from *B. subtilis* 168 **(A)** and LytA, LytB, LytC, and LytD from *L. plantarum* WCFS1 **(B)** Protein size (aa) corresponds to the precursor with its exportation signal-peptide. SH3b-like domain structures were predicted by Phyre 2.0 (www.sbg.bio.ic.ac.uk/phyre2/). **(C)** Alignment of conserved regions of NlpC/P60 domains that contain the essential residues for catalysis (Cys, His, Asp; boxed). NlpC/P60 endopeptidases of *B. subtilis* and *L. plantarum* are indicated in black and red, respectively. **(D)** Conserved regions between *L. plantarum* LytC, *B. subtilis* YkfC (YkfC_Bsu_), and *B. cereus* YkfC (YkfC_Bce_) that are involved in the selection of PG stem peptides ending with L-Ala. Key residues involved in substrate selection (Tyr and Asp) are boxed. Alignments in **(C,D)** were obtained with PRALINE (http://www.ibi.vu.nl/programs/pralinewww/).

In the last family, the NlpC/P60 D,L-endopeptidases deserves a special interest in Gram-positive bacteria since they were shown to have a major role in morphogenesis and cell cycle of *Bacillus subtilis*. In this species, seven NlpC/P60 endopeptidases were identified (Figure 1A). With the exception of PgdS that is strictly dedicated to the hydrolysis of poly-γ-glutamate, all the other D,L-endopeptidases were shown to hydrolyze PG (i.e., LytE, LytF, CwlS, CwlO, and CwlT) or PG fragments for their potential recycling (i.e., YkfC) (Schmidt et al., 2001; Fukushima et al., 2018). All of them were shown to display a γ-D-Glu-*meso*-DAP muropeptidase activity (Ishikawa et al., 1998; Margot et al., 1999; Schmidt et al., 2001; Yamaguchi et al., 2004; Fukushima et al., 2006, 2007). With the exception of the bifunctional CwlT enzyme that is involved in the conjugation of ICE*Bs1* (Fukushima et al., 2007), LytE, LytF, CwlS, and CwlO are modular enzymes implicated in morphogenesis (Hashimoto et al., 2012). CwlO and LytE, whose co-inactivation is synthetically lethal, are required for cell elongation (Hashimoto et al., 2012). However, they perform specific roles and they are differentially controlled by players of the elongation machinery (Domínguez-Cuevas et al., 2013; Meisner et al., 2013). Inactivation of CwlO leads to slightly bent and wider cells than the wild type while inactivation of LytE leads to slightly longer and thinner cells (Domínguez-Cuevas et al., 2013; Meisner et al., 2013). Besides its role in cell elongation, LytE was also reported to play a role in cell separation (Carballido-López et al., 2006). In addition, CwlO, which contains two coiled-coil domains, is activated by the membrane protein complex FtsEX (Domínguez-Cuevas et al., 2013; Meisner et al., 2013), while LytE, which contains three LysM PG-binding domains, was proposed to be guided by the actin-like cytoskeleton protein MreBH (Carballido-López et al., 2006; Domínguez-Cuevas et al., 2013; Meisner et al., 2013). Concerning the two last D,L-endopeptidases, LytF and CwlS, which contain five and four LysM domains, respectively, they were shown to be strictly implicated in the cell separation process (Yamamoto et al., 2003; Fukushima et al., 2006).

*Lactobacillus plantarum* is a commensal Gram-positive bacterium of the human gastrointestinal tract, which displays a *B. subtilis*–like rod shape (Kleerebezem et al., 2010). As a non-sporulating bacterium, *L. plantarum* only harbors 12 predicted PGHs, while *Bacillus subtilis* genome encodes at least 35 PGHs (Smith et al., 2000; Rolain et al., 2012). The predicted autolysome of *L. plantarum* contains 2 glucosaminidases (Acm1 and Acm2), 2 muramidases (Lys1 and Lys2), 3 lytic transglycosidases (MltA, MltB, MltC), 1 amidase (LytH), and 4 D,L-endopeptidases of the NlpC/P60 family (LytA, LytB, LytC, and LytD) (Rolain et al., 2012). In a previous work, we have investigated the functional role of these PGHs through systematic gene inactivation (Rolain et al., 2012). Knockout mutants of 9 out of the 12 predicted PGHs were successfully obtained (Rolain et al., 2012). Two PGHs, Acm2 and LytA, were shown to be differentially involved in the cell cycle. The glucosaminidase Acm2 is responsible of cell separation at a post-divisional stage, while the predicted D,L-endopeptidase LytA was identified as a morphogenic PGH given that its loss resulted in round cells (Rolain et al., 2012). Besides LytA, the function of the three other putative D,L-endopeptidases remain mostly unexplored (Figure 1B; Rolain et al., 2012). By comparison with the modular organization of *B. subtilis* D,L-endopeptidases, LytC and LytD display a similar organization as YkfC with SH3 PG-binding domains, while LytA and LytB possess interesting features such as the absence of coiled-coil domains, a limited number of LysM domains, and the presence of a glycosylated domain rich in alanine, serine, threonine (AST) (Figure 1B). Compared to *B. subtilis*, these specificities suggest that they could have different functions and/or be differently regulated.

The aim of this work is to dissect the role of the four Lyt endopeptidases of *L. plantarum*. To this end, cell morphogenesis was carefully examined in simple mutants of each individual *lyt* gene and double mutants of paralogous genes (i.e., *lytA lytB* and *lytC lytD*). While LytC and LytD did not seem to contribute to cell morphogenesis, LytA and LytB are key morphogenic PGHs. LytA is required for cell elongation, while LytB plays a role in the timing of cell division. Both PGHs are needed for the correct positioning of the division site and display a synthetic growth defect when co-inactivated.

## Materials and Methods

### Strains, Plasmids, and Growth Conditions

Strains and plasmids used in this work are listed in Table 1. Plasmid constructions were performed in *Escherichia coli* (strains AbleK and DH5α). Functional study of Lyt enzymes was performed in *L. plantarum* NZ7100. *E. coli* strains were grown at 37°C with shaking in LB (Lysogeny Broth) medium and *L. plantarum* strains were grown at 30°C in MRS broth (Difco). When appropriate, antibiotics were added to the medium. Chloramphenicol was used at the concentration of 10 μg ml^−1^ for *E. coli* and *L. plantarum*; and erythromycin was used at the concentration of 250 μg ml^−1^ for *E. coli* and 12.5 μg ml^−1^ for *L. plantarum*.

**Table 1:** Strains and plasmids used in this study.

**Strain/Plasmid**	Characteristic(s)^*^	**Reference/source**
**STRAINS**		
***Lactobacillus plantarum***		
WCFS1	Single isolate of strain NCIMB8826	Kleerebezem et al., 2003
NZ7100	WCFS1 *lp_0076::nisRK*	Serrano et al., 2007
TR0015	NZ7100, *lytB::lox72* (Δ*lytB*)	Rolain et al., 2012
TR006	NZ7100, Cm^R^, *lytA::lox66-*P_32_*-cat-lox71* (Δ*lytA*)	Rolain et al., 2012
TR0016	NZ7100, *lytD::lox72* (Δ*lytD*)	Rolain et al., 2012
MCD202	NZ7100, Cm^R^, *lytA::*pGIMCD202, P*_*nisA*_*-*lytA*	This work
MCD20215	TR0015, Cm^R^, *lytA::*pGIMCD202, Δ*lytB* P*_*nisA*_*-*lytA*	This work
MCD203	NZ7100, Cm^R^, *mreB1::*pGIMCD203, P*_*nisA*_*-*mreB1CD*	This work
MCD206	NZ7100, Cm^R^, *lytC::*pGIMCD206, P*_*nisA*_*-*lytC*	This work
MCD208	NZ7100, Cm^R^, *lytC::*pGIMCD208, LytC^−^	This work
MCD20616	TR0016, Cm^R^, *lytC::*pGIMCD206, Δ*lytD* P*_*nisA*_*-*lytC*	This work
***Streptococcus thermophilus***		
LMD-9	Wild type	American Type Culture Collection
***Escherichia coli***		
AbleK	Cloning host, *lac (LacZω-) [Kan^*r*^ McrA^−^ McrCB^−^ McrF^−^ Mrr^−^ HsdR (rK^−^ mK^−^)] [F' proAB lacI^*q*^ ZΔM15 Tn10 (Tet^*r*^)]*, decrease of the copy number of *colE1* plasmids	Stratagene
DH5α	Cloning host, F^−^*φ80 lacZ*ΔM15 Δ(*lacZYA-argF*)U169 *endA1 recA1 hsdR17* (rk^−^ mk^+^) *phoA supE44 thi-1 gyrA96 relA1* λ	Thermo Fisher Scientific
**PLASMIDS**		
**pSIP409 Derivatives**		
pSIP409	Erm^R^, low-copy expression vector, *rep256, colE1*, P*_*orfX*_::gusA*	Sørvig et al., 2005
pSIP103-104	Erm^R^, rep256, *colE1, gusA*, intergenic region between *lp0103* and *lp0104*	Desguin et al., 2015
pGIMCD101	Erm^R^, *rep256, colE1, comR*-P*_*comS*_*-*gusA*	This work
pGIMCD102	Erm^R^, *rep256, colE1, comR*-T*_*ldhL*_*-‘*gusA*	This work
pGIMCD106	Erm^R^, *rep256, colE1, comR*-T*_*ldhL*_-*P*_*shp*0064_*-*gusA*	This work
pGIMCD107	Erm^R^, *rep256, colE1, comR*-T*_*ldhL*_*-P*_*shp*0064_*-MCS	This work
pGIMCD110	Erm^R^, *rep256, colE1, comR*-T*_*ldhL*_*-P*_*shp*0064_*-*ftsZ*-*gfp^+^*	This work
pGIMCD113	Erm^R^*, rep256, colE1, comR*-T*_*ldhL*_*-P*_*nisA*_*-*gfp^+^*	This work
pGIMCD115	Erm^R^, rep256, *colE1, comR*-T*_*ldhL*_*-P*_*shp*0064_*-MCSbis	This work
pGIMCD116	Erm^R^, *rep256, colE1, comR*-T*_*ldhL*_*-P*_*nisA*_-ftsZ-gfp^+^*	This work
pGIMCD117	Erm^R^, *rep256, colE1, comR*-T*_*ldhL*_*-P*_*shp*0064_-lytA*	This work
pGIMCD118	Erm^R^, *rep256, colE1, comR*-T*_*ldhL*_*-P*_*shp*0064_-lytB*	This work
pGIMCD121	Erm^R^, *rep256, colE1, comR*-T*_*ldhL*_*-P_*shp*0064_-lytA^*^	This work
pGIMCD122	Erm^R^, *rep256, colE1, comR*-T*_*ldhL*_*-P*_*shp*0064_-lytAΔLysM*	This work
pGIMCD128	Erm^R^, *rep256, colE1, comR*-T*_*ldhL*_*-P*_*shp*0064_-lytAΔAST*	This work
pGIMCD125	Erm^R^, *rep256, colE1, comR*-T*_*ldhL*_*-P*_*shp*0064_-lytAΔNlpC/P60*	This work
pGIMCD123	Erm^R^, *rep256, colE1, comR*-T*_*ldhL*_*-P*_*shp*0064_-lytA'-NlpC/P60_*LytB*_*, swapping of NlpC/P60 domain, LytA-NlpC/P60_LytB_	This work
pGIMCD132	Erm^R^, rep256, *colE1, comR*-T*_*ldhL*_*-P*_*shp*0064_-lytBΔNlpC/P60*	This work
pGIMCD124	Erm^R^, *rep256, colE1, comR*-T*_*ldhL*_*-P*_*shp*0064_-lytB'-NlpC/P60_*LytA*_*, swapping of NlpC/P60 domain, LytB-NlpC/P60_LytA_	This work
**pUC18Cm Derivatives**		
pUC18Cm	Cm^R^, *colE1*	V. Ladero, laboratory collection
pGIMCD202	Cm^R^, *colE1*, T*_*ldhL*_*-P*_*nisA*_*-*lytA'*	This work
pGIMCD203	Cm^R^, *colE1*, T*_*ldhL*_*-P*_*nisA*_*-*mreB1'*	This work
pGIMCD206	Cm^R^, *colE1*, T*_*ldhL*_*-P*_*nisA*_*-*lytC'*	This work
pGIMCD208	Cm^R^, *colE1*, ‘*lytC'*	This work
**pGIM008 Derivatives**		
pGIM008	Cm^R^, pACYC184 derivative	Palumbo et al., 2006
pGIMCD700	Cm^R^, pGIM008 derivative, T*_*ldh*_*	This work
pGIMCD702	Cm^R^, pGIM008 derivative, T*_*ldh*_*-P*_*nisA*_*-*lytA'*	This work
pGIMCD703	Cm^R^, pGIM008 derivative, T*_*ldh*_*-P*_*nisA*_*-*mreB1'*	
pGIMCD706	Cm^R^, pGIM008 derivative, T*_*ldh*_*-P*_*nisA*_*-*lytC'*	This work
**pNZ8048 Derivatives**		
pNZ8048	Cm^R^, P*_*nisA*_*	Kuipers et al., 1997
pGIMCD301	Cm^R^, P*_*nisA*_*-*gfp^+^*	This work

* *Cm^R^ and Erm^R^, resistance to chloramphenicol and erythromycin, respectively*.

### Nisin and ComS Induction

Nisin A (Sigma Aldrich) was used to induce P_*nisA*_ in the conditional mutants. Conditional mutant strains were cultured overnight in presence of nisin 25 ng ml^−1^. Then, after dilution at an OD_600_ of 0.1, strains were either not induced or induced with nisin (1 or 25 ng ml^−1^).

The ComS-inducible system was used for complementation assays and localization of the FtsZ ring (fusion FtsZ-GFP^+^). ComS from *S. thermophilus* (LPYFAGCL) was synthesized by Peptide 2.0 (Chantilly, VA, USA). The dehydrated peptide was suspended in sterile milliQ water at a stock concentration of 100 μM. Complementation strains were diluted at an OD_600_ of 0.1, induced 2 h later with 8 μM of ComS, and observed between 1 h 30 min and 6 h after induction. Strains producing FtsZ-GFP^+^ were diluted at an OD_600_ of 0.1, induced 2 h later as reported above, and shaken for improving GFP^+^ maturation until their observation 2 h later.

### Growth Monitoring

Bacteria were cultured overnight in MRS supplemented with antibiotics and inducers when needed, diluted at an OD_600_ of 0.05 in the same medium, and separated in 96-wells plates. The growth was monitored in a multi-plate reader Infinity Pro-200 (Tecan) every 10 min at 600 nm during 12 h.

### Microscopy Observations

Cells were collected in exponential phase from MRS cultures (with antibiotics and inducers when needed) and resuspended in PBS buffer. Bacteria were observed on agarose pads composed of 1% agarose PBS buffer for static observations and of 1% agarose MRS for time-lapse observations. Cellular membranes were stained with FM4-64 (Life Technologies) as reported before (Andre et al., 2011). Images were obtained using an Axio I inverted microscope (Zeiss) equipped with an α Plan-Apochromat objective (100 × /1.46 Oil DIC M27) (Zeiss), a HXP 120 C lighting unit (Zeiss) and C10600 ORCA-R2 camera (Hamamatsu). The fluorescence of FM4-64 and FtsZ-GFP^+^ was respectively detected with filter sets Cy3 (43 HE) and GFP (38 HE), displaying bandpass excitation (nm): 550/25 (Cy3) or 470/40 (GFP) and bandpass emission: 605/70 (Cy3) or 525/50 (GFP) (Zeiss). Images were analyzed using Axiovision 4.8 (Zeiss), MicrobeTracker (Sliusarenko et al., 2011), or MicrobeJ (Ducret et al., 2016).

### Profiling of Muropeptides

PG from *L. plantarum* strains was prepared by treating a bacterial pellet with SDS, nucleases, and proteases solutions in order to eliminate all the cellular components except PG, according to a protocol previously described (Courtin et al., 2006). This protocol was slightly modified by applying DNase (50 μg ml^−1^) and RNase (50 μg ml^−1^) treatments before hydrofluoric acid extraction. PG was digested with mutanolysin from *Streptomyces globisporus* (Sigma-Aldrich). The resulting muropeptides were analyzed by RP-HPLC as previously reported (Courtin et al., 2006). Muropeptides were identified according to their retention times by comparison to the previously published reference chromatogram for *L. plantarum* PG (Bernard et al., 2011a). In addition, disaccharide-dipeptide (Di) purified from *Lactobacillus casei* (Regulski et al., 2012) was used as standard. The relative abundance (in %) of Di (with or without *O*-acetylation) was calculated as the ratio of the areas of the two peaks over the sum of the areas of all the identified peaks on the chromatogram.

### DNA Manipulations and Transformation

Classical methods of molecular biology were used as previously described (Sambrook et al., 1989). Preparation of electro-competent cells and transformation of *E. coli* and *L. plantarum* were performed as previously reported (Dower et al., 1988; Josson et al., 1989). The Phusion High Fidelity polymerase (NEB) was used for amplification by PCR of inserts used for plasmid constructions. PCR amplifications for validation were performed with the GoTaq polymerase (Promega) in a GeneAmp PCR system (Applied Biosystem). Primers were synthesized by Eurogentec (Belgium) and are listed in Supplementary Table 1.

### General Strategy for the Construction of Plasmids and Mutant Strains

All expression and disruption plasmids were constructed in *E. coli* before their transfer by electroporation in *L. plantarum*. The correct construction of the vectors was validated by PCR (validation primers in Supplementary Table 1) and enzymatic digestion. DNA sequencing was performed to validate the final product (validation primers in Supplementary Table 1). Suicide vectors for gene inactivation were recombined by simple homologous recombination into the chromosome of *L. plantarum* as previously described (Palumbo et al., 2004). The conditional mutants contained a 3′-end truncated copy of the targeted gene (inactive, truncation of the NlpC/P60 domain for LytA and LytC) and a second intact copy under the control of the nisin-inducible promoter. The validation of mutant strains was performed by PCR amplification of regions flanking the site of vector integration and sequencing of the PCR product (validation primers in Supplementary Table 1).

Construction details of plasmids for conditional/disruption mutants, for complementation, for ComS-inducible expression, and for expression of truncated/hybrid proteins and FtsZ-GFP^+^ fusion are presented in Supplementary Text.

## Results

### *In silico* Analysis of NlpC/P60 Endopeptidases From *L. plantarum*

The four D,L-endopeptidases of *L. plantarum* can be separated in two groups based on their modular organization (Figure 1B). The first group contains LytA (Lp_3421, 370 aa) and LytB (Lp_2162, 496 aa). In their mature form, these two enzymes contain LysM domain(s) at the N-terminus; a low complexity AST central domain; and a catalytic domain of the NlpC/P60 family (pfam00877) at the C-terminus. The second group is composed of LytC (Lp_2520, 297 aa) and LytD (Lp_1242, 243 aa). These two proteins contain putative SH3b domains in their N-terminal part that were identified by a structural prediction (Phyre 2.0) and an NlpC/P60 catalytic domain at their C-terminus.

All members of the NlpC/P60 family contain three conserved residues involved in catalysis: Cys, His and a polar residue (His, Asp or Gln) (Anantharaman and Aravind, 2003). The alignment of the NlpC/P60 domains of the four *L. plantarum* Lyt enzymes and the 7 *B. subtilis* D,L-endopeptidases showed that Cys and His residues are fully conserved, as well as the third polar residue that is an Asp in this case (Figure 1C). The 3D structure prediction of the four Lyt catalytic domains suggests that the three conserved catalytic residues are in close proximity (Supplementary Figure 1A), consistent with their contribution to catalysis. Interestingly, LytC and LytD displayed a modular organization, which is similar to YkfC of *B. subtilis* and *Bacillus cereus* (Xu et al., 2010, 2015). Although YkfC of *B. subtilis* has been proposed to be intracellular due to the absence of a predicted signal-sequence, the *B. cereus* enzyme is predicted to be extracellular (Xu et al., 2010), as for LytC and LytD. YkfC is a γ-D-Glu-*meso*-DAP endopeptidase specific for PG peptides with free N-terminal L-Ala, suggesting a role in PG peptide recycling (Schmidt et al., 2001; Xu et al., 2010). Some NlpC/P60 endopeptidases were identified as recycling enzymes based on the presence of two conserved residues: an aspartate in their active site and a tyrosine at the junction between catalytic and SH3 PG-binding domains, which both restrict the access of the catalytic site to short PG peptides (Xu et al., 2015). Interestingly, LytC is the sole Lyt enzyme that contains these two conserved residues (Figure 1C). We also examined the phylogenetic proximity of NlpC/P60 domains between the D,L-endopeptidases of *L. plantarum* and *B. subtilis* (Supplementary Figure 1B). Interestingly, LytC clustered with YkfC while LytD clustered with the conjugation-related CwlT autolysin. In addition, the NlpC/P60 domains of LytA and LytB form a separate group, while LytE, LytF, CwlS, and CwlO involved in morphogenesis cluster altogether (Supplementary Figure 1B).

Finally, the accessory domains of LytA and LytB display interesting features compared to the morphogenic D,L-endopeptidases of *B. subtilis*. Both enzymes contain a central AST domain of a different size (186 and 250 aa, respectively), which is predicted to be unstructured. Both domains were shown to be *O*-glycosylated, suggesting a regulation mechanism of the enzyme activity or stability (Fredriksen et al., 2013; Rolain et al., 2013). LytA and LytB also contain a lower number of LysM domains (i.e., 1 or 2) compared to the morphogenic endopeptidases of *B. subtilis* (i.e., 3–5) (Figures 1A,B). The LysM domain is a well-established PG-binding domain in PGHs that recognizes the GlcNAc – X – GlcNAc motif of PG glycan strands (Visweswaran et al., 2011; Mesnage et al., 2014). Interestingly, the LysM domains from LytA and LytB have different isoelectrical points (IP). The single LysM of LytA is basic (IP of 8.34) as found for LysM domains of *B. subtilis* D,L-endopeptidases (IP comprised between 9.22 and 10.14) while the two LysM of LytB are in the acidic range (IPs of 5.43 and 5.18). As the ability of LysM domains to bind PG is linked to their IP (Visweswaran et al., 2011), this could indicate that LytA and LytB bind PG under different pH conditions.

### LytC and LytD Are Not Involved in Morphogenesis

In the first part of this work, we investigated the role of the putative D,L-endopeptidases LytC and LytD. A conditional mutant containing a chromosomal copy of the *lytC* gene under the control of the nisin-inducible promoter (P_*nisA*_-*lytC* fusion) was constructed. Growth and cell morphology of the mutant strain was monitored in the absence of the nisin inducer (N0). The mutant strain did not show any obvious difference compared to the WT (strain NZ7100) (Supplementary Figure 2). Similar results were obtained for a mutant in which the chromosomal copy of *lytC* was disrupted by single crossover with a suicide plasmid carrying an internal fragment of the gene (data not shown). The stable deletion of *lytD* (Δ*lytD*) has previously been obtained (Rolain et al., 2012). The phenotype of the mutant strain was reinvestigated regarding growth and cell morphology. As previously reported, it was indistinguishable from the WT (Supplementary Figure 2; Rolain et al., 2012). Since LytC and LytD may have redundant functions due to their similar organization (Figure 1B), a double *lytC lytD* mutant was also constructed. Again, this mutant grown in absence of nisin behaved as the WT (Supplementary Figure 2).

Altogether, these data indicate that LytC and LytD are not involved in *L. plantarum* morphogenesis under the tested conditions.

### LytA Is a Major Peptidoglycan Hydrolase in Cell Morphogenesis

**Figure 2:**
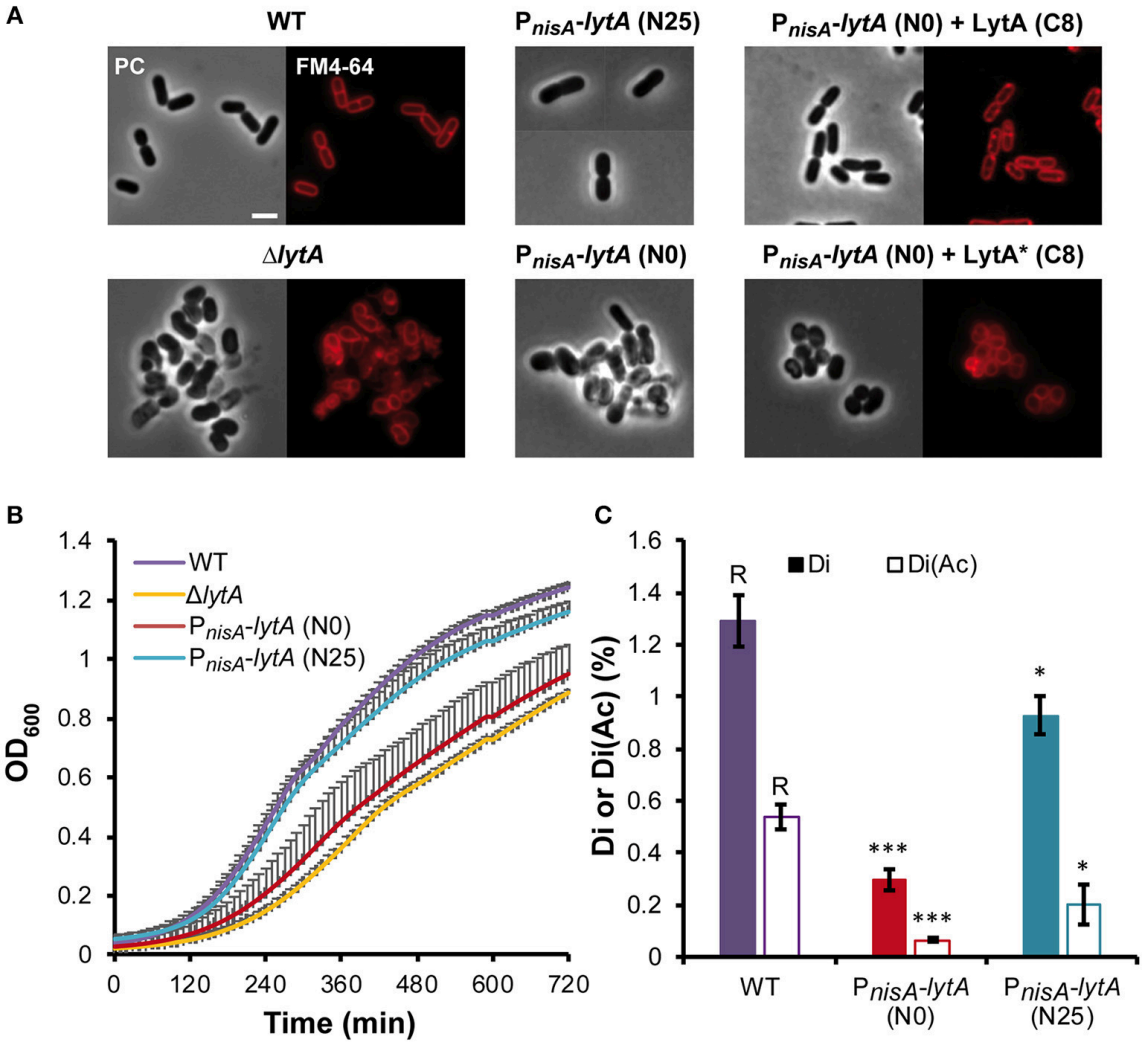
Effect of LytA deficiency on cell morphology, growth, and PG composition. **(A)** Images of *L. plantarum* cells obtained by phase contrast (PC) microscopy and epifluorescence microscopy for membrane labeling with FM4-64. Left panel, WT and Δ*lytA* mutant; middle panel, conditional P_*nisA*_-*lytA* mutant without (N0) or with nisin 25 ng ml^−1^ (N25); right panel, complementation of P_*nisA*_-*lytA* mutant with LytA (P_*shp*0064_-*lytA*; + LytA) and a catalytic mutant of LytA (P_*shp*0064_-lytA*; + LytA*), grown without nisin (N0) in presence of ComS (8 μM, C8). Cells were collected in exponential phase from MRS cultures (with chloramphenicol when needed) and observed on agarose pads after suspension in PBS. Similar observations were obtained from at least 3 independent experiments. The scale bar is 2 μm. **(B)** Growth curves of WT, Δ*lytA* mutant, and P_*nisA*_-*lytA* mutant (N0 and N25) in MRS medium. Curves were generated from triplicates (mean values + standard deviations). **(C)** Percentage of disaccharides-dipeptides without and with *O*-acetylation (Di and Di+Ac, respectively) in the PG of WT and P_*nisA*_-*lytA* mutant (N0 and N25) after mutanolysin digestion. Mean values of three independent extractions ± standard deviations. Significance with respect to the WT (R, reference) is based on Student's *t*-test. *P < 0.05 and ***P < 0.001, respectively.

We previously reported the construction of a stable *lytA* null mutant (Δ*lytA*) (Rolain et al., 2012). The cell morphology of this mutant was strongly affected, with round and aggregated cells instead of rod-shaped and well-separated cells observed for wild type (WT) cells (Figure 2A). In addition, this mutant displayed a growth defect and cannot be transformed to perform further analyses such as complementation studies (Rolain et al., 2012). For these reasons, we decided to construct a conditional *lytA* mutant by placing the chromosomal copy of *lytA* under the control of P_*nisA*_ (P_*nisA*_-*lytA* fusion). In absence of nisin (N0), cell morphology and growth of this conditional mutant strain was similar to the stable Δ*lytA* mutant (Figures 2A,B). Under nisin induction (25 ng ml^−1^, N25), the strain recovered a WT phenotype regarding growth and morphology, except for a slightly larger diameter (Figures 2A,B).

The conditional mutant strain was complemented by a copy of *lytA* expressed under the control of the ComS-inducible system (P_*shp*0064_-*lytA* fusion) carried by a low copy number plasmid (see Supplementary Text for details on the ComS-inducible system). In the presence of the inducer peptide ComS (8 μM; C8), the LytA-depleted strain recovered the WT rod-shaped morphology (Figure 2A). In addition, we showed that LytA activity is crucial since complementation with a catalytic inactive mutant of LytA [Cys_284_ to Ala (Figure 1C); named LytA^*^] was unable to restore the WT rod-shaped morphology (Figure 2A).

Previous analyses of PG extracted from the Δ*lytA* mutant suggested that LytA was cleaving the bond between D-Glu and *meso*-DAP in the peptide side chains of the PG network (Rolain et al., 2012). To further correlate the observed morphological phenotypes with LytA activity, analyses of PG composition were performed in triplicates for the conditional mutant strain (P_*nisA*_-*lytA*) in presence or absence of nisin (Figure 2C, Supplementary Figure 3, and Supplementary Table 2). PG analysis of the WT strain showed that the pool of disaccharides with two amino acids [GlcNAc-MurNAc-L-Ala-D-Glu with and without *O*-acetylation, (Di and Di+Ac, respectively)], which are potential products of D,L-endopeptidase activity on PG stem peptides, represents 1.83% of total soluble muropeptides. For the nisin-depleted strain (N0), the combined amount of Di and Di+Ac was 5-fold lower (0.37%), consistent with a lower production of LytA. When the conditional mutant strain was cultured in the presence of nisin (25 ng ml^−1^, N25), the combined amount of Di and Di+Ac was 3-fold higher (1.13%) than in non-induced conditions, reaching a level close to the WT but remaining slightly lower. These data strongly suggest that LytA is a γ-D-Glu-*meso*-DAP endopeptidase that cleaves the bond between D-Glu and *meso*-DAP in the PG meshwork, and that its absence or decreased activity significantly alter the PG structure.

Altogether, these results demonstrate that LytA is a major PGH of *L. plantarum* morphogenesis and that its endopeptidase activity, more than its physical presence in a PG biosynthetic complex, is required for proper growth and cell cycle progression.

### LytA Is Required for Cell Elongation and Septum Positioning

**Figure 3:**
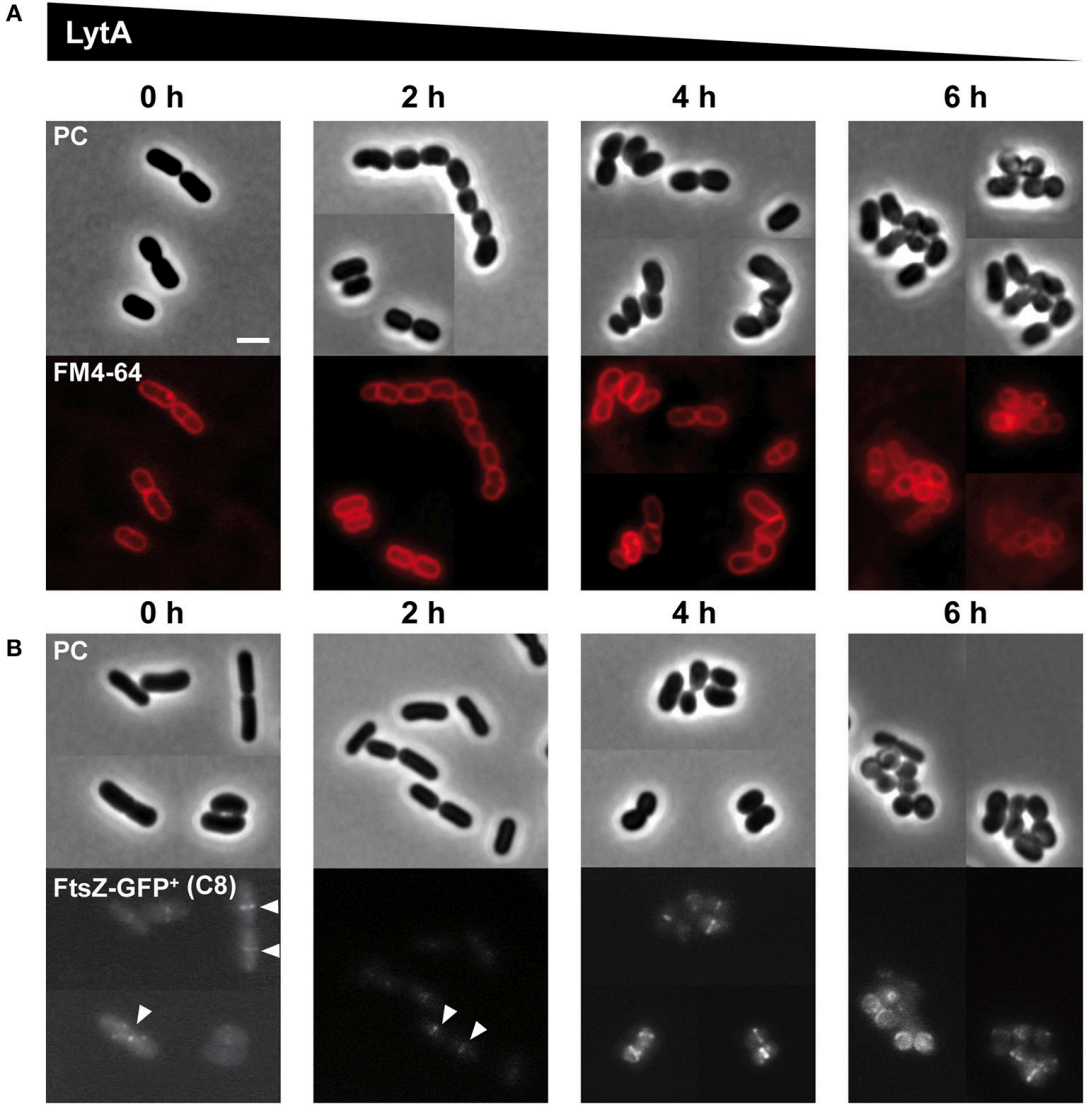
Effect of progressive LytA depletion on cell morphology and division site positioning. **(A)** P_*nisA*_-*lytA* mutant cells observed during nisin depletion (0, 2, 4, 6 h) by phase contrast (PC) microscopy and FM4-64 staining. The scale bar is 2 μm. **(B)** P_*nisA*_-*lytA* mutant cells expressing an FtsZ-GFP^+^ fusion (P_*shp*0064_-*ftsZ-fgp*^+^; + FtsZ-GFP^+^) observed during nisin depletion (0, 2, 4, 6 h) by phase contrast (PC) microscopy and epifluorescence microscopy for the localization of FtsZ-GFP^+^. White arrows indicate Z-rings in normal rod-shaped cells. Bacteria were cultured in MRS with erythromycin and chloramphenicol, and induced with ComS (8 μM, C8). Cultures were moderately shaken after ComS induction. The scale bar is 2 μm. For **(A,B)**, similar observations were obtained from at least two independent experiments.

We previously reported that the morphology of the Δ*lytA* mutant was strongly affected with the presence of aggregated round cells of variable diameters and alterations of PG thickness at division sites (Rolain et al., 2012). Here, we used the conditional *lytA* mutant to understand the impact of a progressive depletion of LytA on cell morphogenesis. Cells were first stained with FM4-64 to observe membranes and septum position (Figure 3A) or labeled with an FtsZ-GFP^+^ fusion to visualize the divisional Z rings (Figure 3B). Figure 3 shows the degenerative process of the conditional *lytA* mutant taken at different time points after nisin removal. In early steps (0–2 h), cells remained similar to WT, forming chains of rod-shaped bacteria with division planes and Z rings at mid-cell. After 4 h of depletion, cells formed short chains of 2–3 deformed cells. Dividing cells started to bend and septa seemed to be placed in an abnormal orientation (Figure 3, Supplementary Figure 4A). After prolonged depletion (6 h), a large proportion of cells were round and aggregated. Membrane labeling appeared brighter and less homogenous, which may indicate that membrane biogenesis is altered [Figure 3 (6 h), Supplementary Figure 5A]. In round cells, FtsZ does not seem to be correctly localized, forming dots or arcs at the cell periphery (Figure 3, 6 h). These observations show that LytA is required for cell elongation. In addition, population analysis showed that more than 70% of dividing cells (*n* > 300) have an altered positioning of the division plane after 6 h of LytA depletion (deviation >10% from the median position), while only ~10% of WT cells have slightly misplaced septa (a sample of LytA-depleted cells with misplaced septa is shown in Supplementary Figures 4B, 5A). These asymmetrical division events likely account for the round cells of different diameters (Supplementary Figure 5B) that were previously reported for the stable Δ*lytA* mutant (Rolain et al., 2012). Mispositioning of division sites might be due to an indirect effect resulting from the lack of LytA-mediated PG remodeling activity, or from the absence of elongation.

**Figure 4:**
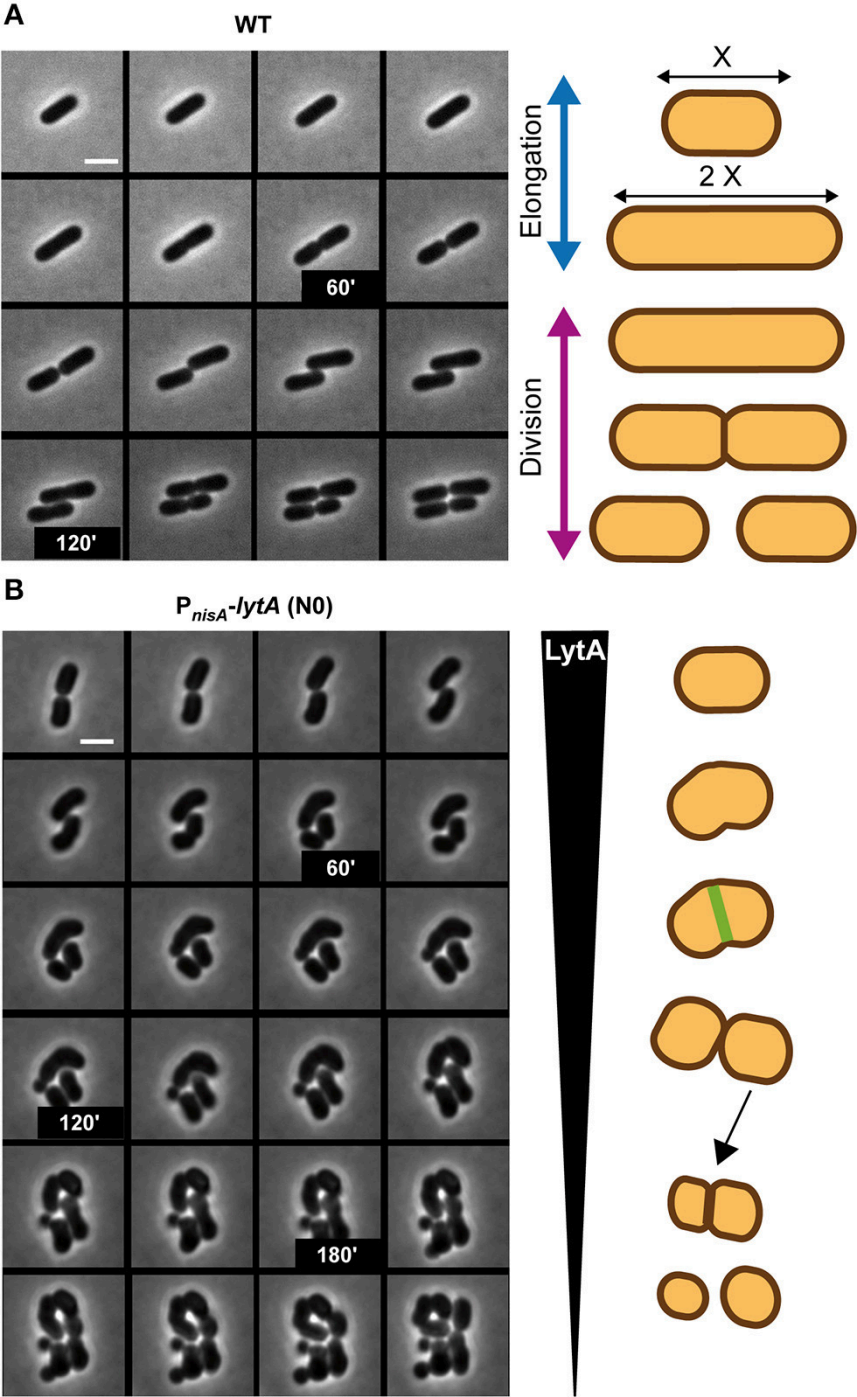
Comparison of the cell cycle of WT and LytA-depleted strain. **(A)** Time-lapse microscopy of the WT strain. **(B)** Time-lapse microscopy of the P_*nisA*_-*lytA* mutant strain without nisin (N0). The cell cycle of both strains is schematically represented on the right. Bacteria were grown on MRS-containing agarose pads at 30°C. The scale bar is 2 μm. For **(A**,**B)** one representative time lapse from at least two independent experiments.

Morphological alterations during the cell cycle of *lytA*-depleted cells were also followed by time-lapse microscopy. The conditional *lytA* mutant was cultured overnight in the presence of nisin and then diluted on an MRS-containing agarose pad in the absence of the inducer. The cell cycle of the LytA-depleted strain was compared to the cell cycle of the WT strain (Figure 4, Supplementary Movies 1, 2). WT cells were shown to elongate until they reached twice their original length and then divided into two daughter cells of equal length (Figure 4A). For the LytA-depleted strain, cells started as rod-shaped bacteria and progressively reached the round and aggregated morphology after 2–3 generations (Figure 4B, left panel). Before the first division, a short elongation phase was observed but cells never doubled their length. Then, cells started to bend and division occurred like a break in the middle of the cell (V-shaped dividing cells). At the next generation, elongation is inhibited and new daughter cells were generated by asymmetrical division. This resulted in the formation of round cells of different diameters, including mini cells. At the end of the depletion process, only round and aggregated cells were observed due to absence of elongation, misplacement of division sites, and the lack of cell separation.

To get further insight into the role of LytA during the cell cycle, we chose to compare the LytA-depleted phenotype with that obtained from the conditional inactivation of the *mreB1CD* locus whose homolog from *B. subtilis* has been shown to be specifically involved in cell elongation (Formstone and Errington, 2005). To this end, the chromosomal copy of *L. plantarum mreB1CD* operon was placed under the control of the nisin-inducible promoter (P_*nisA*_-*mreB1CD* fusion). The phenotype of the resulting MerB1CD-depleted cells was observed in time lapse (Supplementary Figure 6 and Supplementary Movie 3). From these observations, it appeared that cells became rapidly unable to elongate, giving rise to small spherical cells after one or two division cycles. This phenotype is not exactly the same as that observed for the LytA-depleted mutant in which impairment of elongation was accompanied with a defect in septum positioning. Thus, the data suggest that the lack of elongation for the *lytA* mutant is not, by itself, responsible for the mispositioning of the septum.

Altogether, the results demonstrate that LytA is a crucial player of *L. plantarum* cell cycle required for the elongation process. In addition, its absence has probably an indirect effect on the correct placement of the division site.

### LytB Plays a Key Role in Septum Maturation and Timing of Division

**Figure 5:**
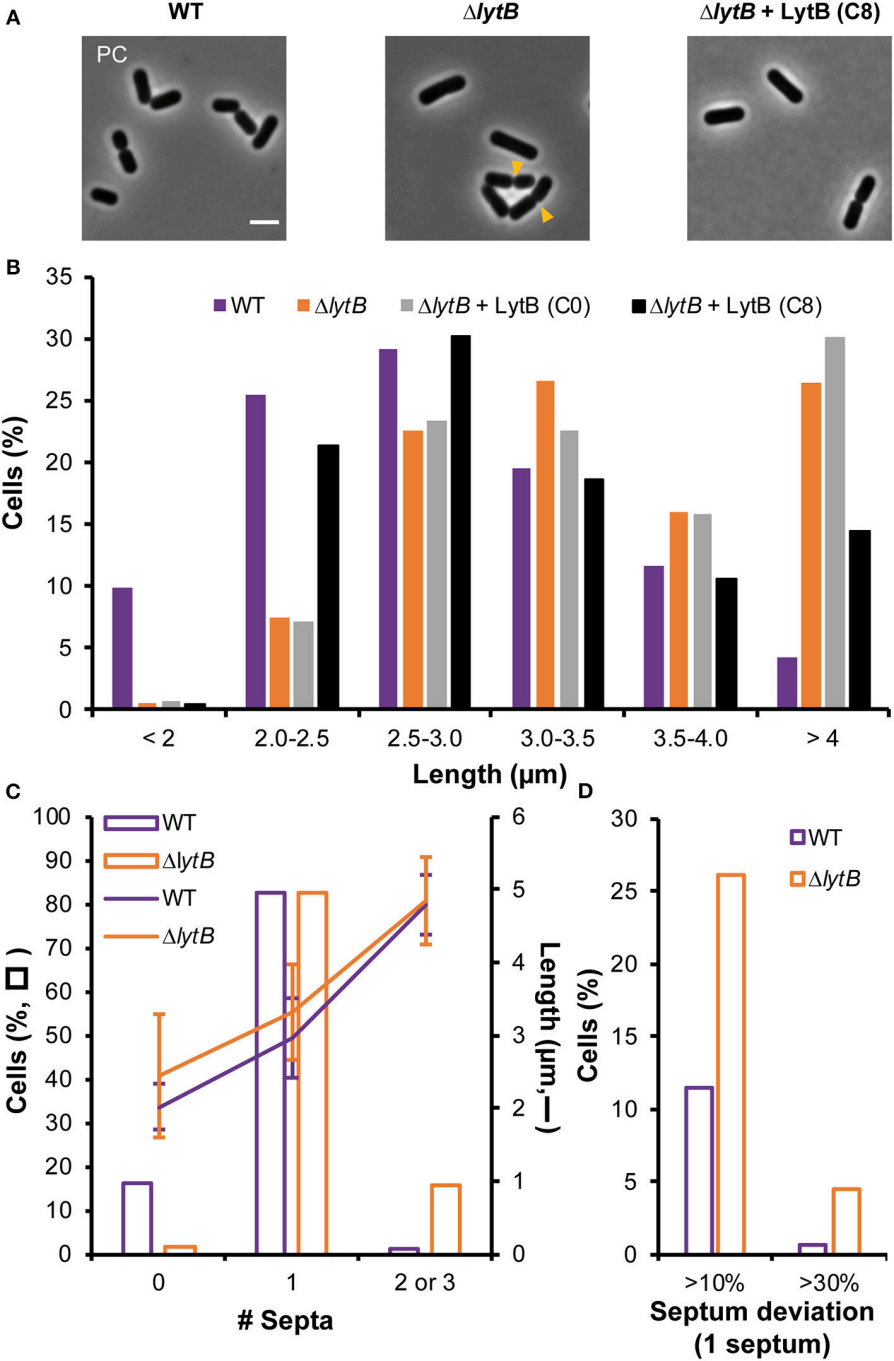
Effect of LytB deficiency and its complementation on cell morphology and division site positioning. **(A)** Images of cells of WT, Δ*lytB* mutant, and Δ*lytB* mutant complemented with LytB (P_*shp*0064_-*lytB*; + LytB) obtained by phase contrast (PC) microscopy. Yellow arrowheads show asymmetrical divisions in Δ*lytB* mutant cells. Bacteria were grown in MRS with erythromycin and ComS (8 μM, C8) when needed. Scale bar is 2 μm. **(B)** Cell length (μm) of WT, Δ*lytB* mutant and Δ*lytB* mutant + LytB without (C0) and with ComS (C8) measured in exponential growth phase. Cells were cultured in MRS with erythromycin and ComS when needed. **(C)** Number of septa (0 to 3; bars) related to cell length (lanes; mean values ± standard deviations) in WT and Δ*lytB* mutant cells. **(D)** Relative septum deviation (% of deviation from the median position of cells stained with FM4-64) in mono-septal cells of WT and Δ*lytB* mutant. For **(B–D)** measures were obtained from triplicates by using MicrobeJ with *n* > 500 cells.

We previously constructed a stable deletion mutant of *lytB* (Δ*lytB*) (Rolain et al., 2012). Growth and overall cell morphology of the mutant were initially reported to be similar to the WT (Figure 5A) (Rolain et al., 2012). However, a more thorough analysis of the cell size revealed that the mutant has a mean cell length significantly longer than the WT [3.5 ± 0.85 vs. 2.8 ± 0.67 μm, respectively; mean values ± standard deviations, *n* > 600 cells from triplicates, Kolmogorov–Smirnov (KS) test, *P* < 0.001], with a larger proportion of cells exceeding 4 μm (6-fold) and a lower proportion of cells shorter than 2 μm (16-fold) (Figure 5B). For the cell diameter, it remained unchanged in comparison to the WT (0.85 ± 0.12 vs. 0.82 ± 0.13 μm). The Δ*lytB* mutant was also complemented by a *lytB* copy under the control of the ComS-inducible system (P_*shp*0064_*-lytB* fusion). In the presence of ComS, the mean cell length of the complemented strain was significantly reduced compared to growth conditions without ComS (3.0 ± 0.80 vs. 3.6 ± 0.70, respectively; *n* > 900 cells from triplicates, KS test, *P* < 0.001). However, the distribution of length frequencies of the complemented strain compared to the WT showed a partial complementation (Figure 5B), suggesting that the amount of LytB is inadequate for a full reversion of the phenotype.

The observed cell length increase in the LytB-deficient strain was correlated with septum positioning using FM4-64 labeling (Figure 5C, Supplementary Figure 7). LytB-deficient cells that are longer than 4 μm contained 2 or 3 septa (Figure 5C). The presence of two septa instead of three in more than 50% of long cells suggests that the formation of septa in daughter cells was not synchronized, while synchronization was observed in the WT (Supplementary Figure 7). In addition, lateral septa in WT daughter cells appeared at the end of the constriction of the median septum, while the maturation of the mid-cell septum seemed delayed in LytB-deficient cells (Supplementary Figure 7). This delayed maturation in dividing cells was confirmed by examining FtsZ positioning (FtsZ-GFP^+^). In WT, Z rings localized in daughter cells at the end of the constriction of the septum of the mother cell while the migration of Z rings in LytB-deficient cells took place at an earlier stage when maturation of the median septum was largely incomplete (Supplementary Figure 8). We also examined the lateral positioning of septa in dividing cells, which was affected in LytB-deficient cells compared to WT cells (Figure 5D, Supplementary Figure 7). In mono-septal cells, lateral mispositioning of the septum (>10% deviation from the median position) was observed in ~25% of mutant cells, including 5% of cells with major misplacement (>30% of deviation) (Figure 5D). In multi-septal cells, a similar situation was observed with numerous lateral misplacements of septa, leading in extreme cases to the production of minicells (Supplementary Figure 7). All these observations suggest that LytB is involved in septum maturation and that its absence affects the timing of division and the lateral positioning of the septum.

**Figure 6:**
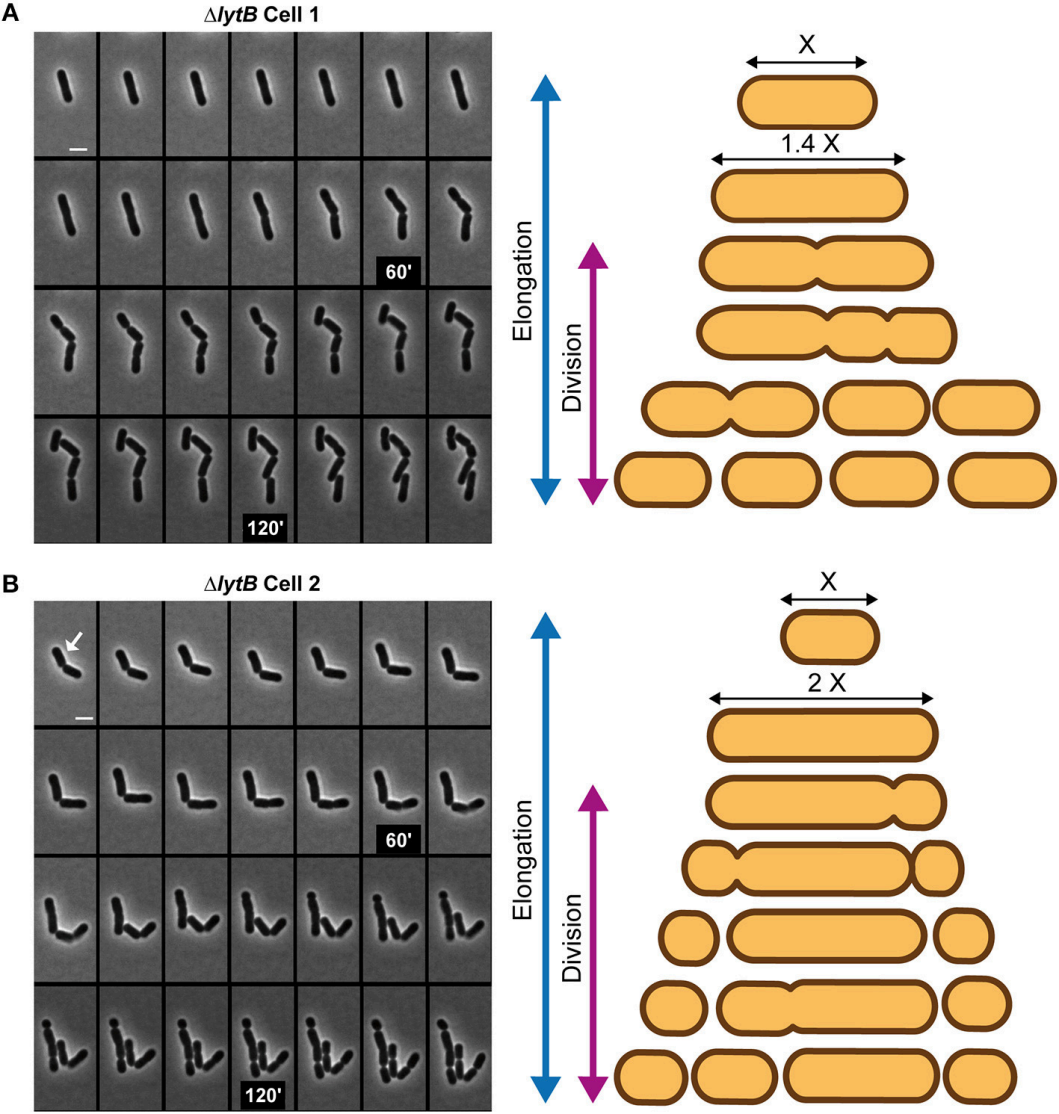
Cell cycle of the LytB-deficient strain. Time-lapse microscopy of Δ*lytB* mutant cells showing a deregulation between elongation and division **(A)** and asymmetrical divisions **(B)**. In **(B)** the selected cell for the scheme is indicated with a white arrow. Scheme of the cell cycle of two selected cells are displayed on the right. Bacteria were grown on MRS-containing agarose pads at 30°C. The scale bar is 2 μm.

To further document the role of LytB in cell-cycle dynamics, the Δ*lytB* mutant was observed by time-lapse microscopy. In Figure 6, two behaviors of Δ*lytB* mutant cells are shown. In the first case (Figure 6A, Supplementary Movie 4), which is representative of the behavior of many cells, we observed that the cell elongated until it reached 6 μm prior to divide, which is 2 μm longer than the WT at the same pre-divisional state (Figure 4A). At the next generation, the daughter cells did not elongate significantly before starting division (Figure 6A). In an alternative rarer scenario, the left cell elongated until 5 μm and then a first asymmetrical division occurred, which gave rise to a mini cell (Figure 6B, Supplementary Movie 5). Then, a second asymmetrical division took place at the opposite pole in the long daughter cell (Figure 6B). From various time-lapse experiments, ~25% of division events were asymmetrical (*n* > 100). In addition, we observed that some small cells stopped to grow and finally lysed, which may explain the lower proportion of short cells (< 2 μm) at the whole population level of the Δ*lytB* mutant (Figure 5B, Supplementary Figure 9).

While elongation and division are well-separated processes in the WT (Figure 4A), the Δ*lytB* mutant is not able to regulate the timing of division during elongation. In addition, LytB deficiency affects the lateral placement of the division site, which could be asymmetrically localized in short or long cells (Figure 6B).

### The Combined Inactivation of LytA and LytB Severely Affects Cell Growth

**Figure 7:**
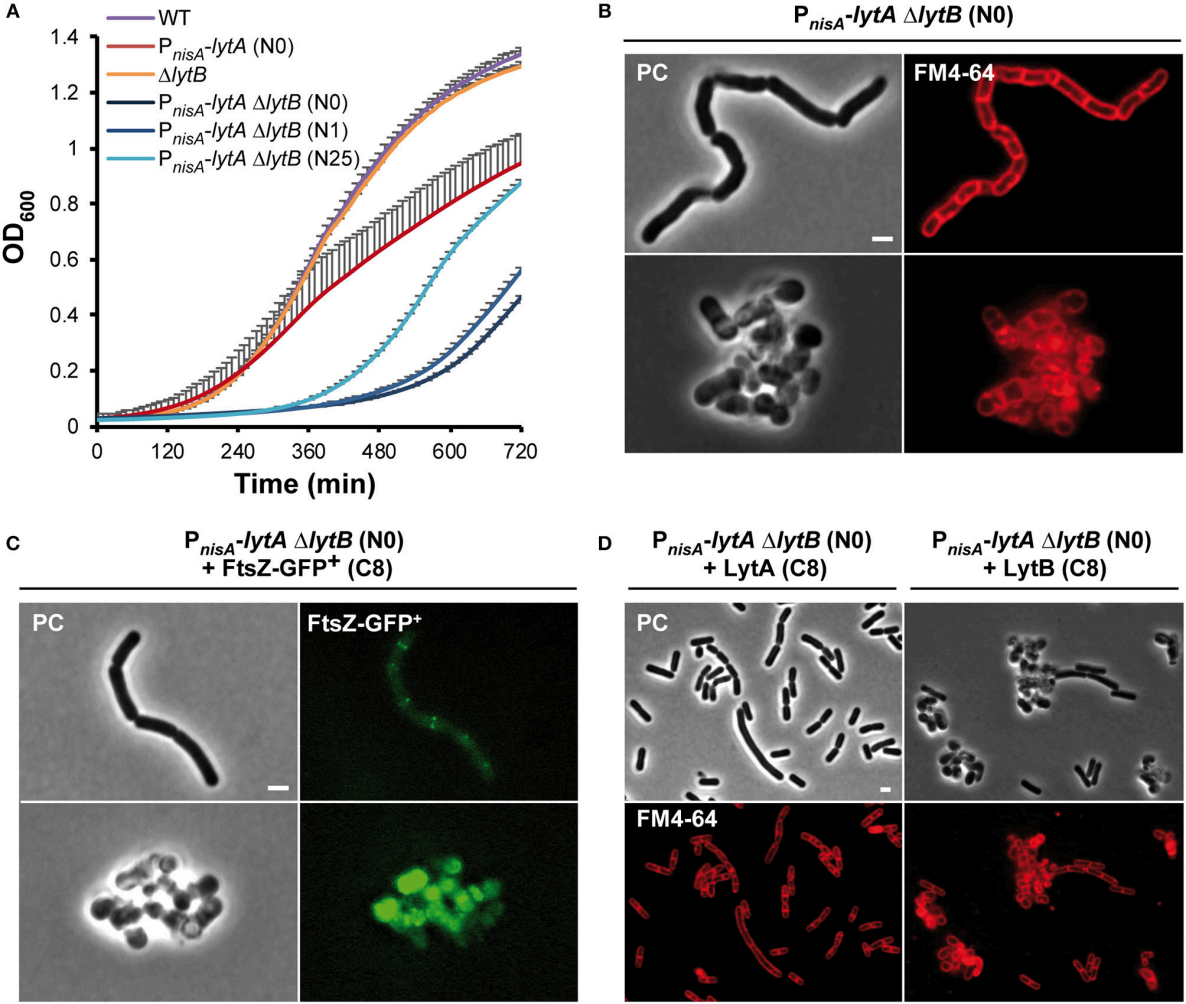
Effect of the double LytA LytB deficiency on growth and cell morphology. **(A)** Growth curves of WT, P_*nisA*_-*lytA* mutant, Δ*lytB* mutant and double P_*nisA*_-*lytA* Δ*lytB* mutant. N0, non-induced; N1 and N25, nisin 1 and 25 ng ml^−1^, respectively. Bacteria were grown in MRS with chloramphenicol and nisin when appropriate. Curves were generated from triplicates (mean values + standard deviations). **(B)** Cells of the double P_*nisA*_-*lytA* Δ*lytB* mutant observed by phase contrast (PC) microscopy and FM4-64 staining under nisin depletion (2 h). Cells were collected from MRS cultures (with chloramphenicol) and observed on agarose pads after suspension in PBS. The scale bar is 2 μm. **(C)** P_*nisA*_-*lytA* Δ*lytB* mutant cells expressing FtsZ-GFP^+^ (P_*shp*0064_-*ftsZ-fgp*^+^; + FtsZ-GFP^+^) observed by epifluorescence microscopy. Bacteria were cultured in MRS with erythromycin and chloramphenicol, and induced with ComS (8 μM, C8) in absence of nisin (N0). Cultures were moderately shaken after induction. The scale bar is 2 μm. **(D)** Cells of P_*nisA*_-*lytA* Δ*lytB* mutant complemented with LytA (P_*shp*0064_-*lytA*; + LytA, left panels) and LytB (P_*shp*0064_-*lytB*; + LytB, right panels) observed by phase contrast microscopy and FM4-64 staining under nisin depletion (2 h). Bacteria were cultured in MRS with erythromycin and chloramphenicol, and induced with ComS (8 μM, C8) in absence of nisin. Scale bar is 2 μm. For **(B–D)** similar observations were obtained from at least three independent experiments.

The above results suggest that LytA and LytB play different roles in the cell cycle of *L. plantarum*. This contrasts with the situation of *B. subtilis* where elongation is controlled by two D,L-endopeptidases (i.e., CwlO and LytE) (Domínguez-Cuevas et al., 2013; Meisner et al., 2013). Since LytB does not appear to be a rescuer of LytA, we decided to construct a double *lytA lytB* mutant to determine to what extent their activities interact with each other. Previous attempts to construct a stable mutant deleted for both *lytA* and *lytB* (Δ*lytB* Δ*lytA*) failed, which was an indirect indication that the combined presence of LytA and lytB is essential for growth (Rolain et al., 2012). To solve this issue, we transferred the P_*nisA*_-*lytA* conditional fusion into the Δ*lytB* mutant. The resulting double mutant (P_*nisA*_-*lytA* Δ*lytB*) was obtained in the presence of nisin. Compared to the singly depleted P_*nisA*_-*lytA* mutant, the double P_*nisA*_-*lytA* Δ*lytB* mutant displayed a severe growth defect (Figure 7A). No growth of the double mutant was observed during the first 6 h while the simple mutant strain started to grow after 2 h post-inoculation (Figure 7A). The retarded growth of the double mutant is likely due to suppressor mutations since the corresponding cells displayed a typical rod-shape morphology (Figure 7A and data not shown). These suppressors are probably resulting from a reversion of the nisin-controlled expression system as previously observed for another conditional mutant of an essential gene (alanine racemase) in *L. plantarum* (unpublished data). So, this result indicates that the combined presence of LytA and LytB is (nearly) essential for growth and reinforces the key role played by both enzymes in PG remodeling during the cell cycle.

The cell morphology of the double mutant was then examined after 2 h of nisin depletion. Intriguingly, a mixed situation was observed with round aggregated cells and long cells forming chains, which is reminiscent of either LytA or LytB deficiency, respectively (Figure 7B). This mixed phenotype is likely due to cell-to-cell variations in the extent of LytA depletion. We also examined the positioning of septa and FtsZ-GFP^+^ in the double mutant under nisin depletion (Figures 7B,C). In the long cells, septa and Z ring-like structures formed perpendicularly to the long axis without being localized at mid-cell. This mispositioning is reminiscent of the abnormalities observed with the simple Δ*lytB* mutant (see Figure 6B). Akin to simple LytA-deficient cells, no clear positioning of FtsZ-GFP^+^ was observed in round cells. To investigate this heterogeneous cell morphology, we performed time-lapse experiments (Supplementary Figure 10 and Supplementary Movies 6, 7). At the beginning of the depletion, cells were long and formed chains as found with the Δ*lytB* mutant. Then, they became twisted before their separation in round or unshaped cells resembling to LytA-deficient cells (Supplementary Figure 10). So, the initial observation of a mixed cell morphology under static conditions likely corresponds to different levels of LytA at the early stages of the depletion process.

In order to validate that LytA and LytB have no overlapping function, the double P_*nisA*_-*lytA* Δ*lytB* mutant was complemented by either *lytA* or *lytB*. When the double mutant was grown in the presence of ComS to express the complementing partner and under nisin depletion condition, *lytA*-complemented cells recovered a rod shape but most cells were longer than WT cells (Figure 7D, left panels); whereas *lytB*-complemented cells remained round and aggregated (Figure 7D, right panels). In addition, complementation assays of the simple P_*nisA*_-*lytA* mutant (N0) by *lytB* or the simple Δ*lytB* mutant by *lytA* did not restore the morphology of WT cells (data not shown). Finally, complementation of the simple P_*nisA*_-*lytA* mutant by chimeric proteins where the catalytic domains (NlpC/P60 domains, see Figure 1B) were swapped between both proteins (i.e., LytA-NlpC/P60_LyB_ and LytB-NlpC/P60_LytA_) revealed that the functionality of the LytA protein mainly relies on its accessory domains (Supplementary Figure 11). Indeed, the LytA-NlpC/P60_LyB_ hybrid restored cell elongation while the LytB-NlpC/P60_LytA_ fusion protein did not (Supplementary Figure 11).

Taken together, these results show that the combined PG hydrolytic activity of LytA and LytB is of major importance for *L. plantarum* growth. They also demonstrate that LytA and LytB play separate and non-redundant functions during the cell cycle.

## Discussion

**Figure 8:**
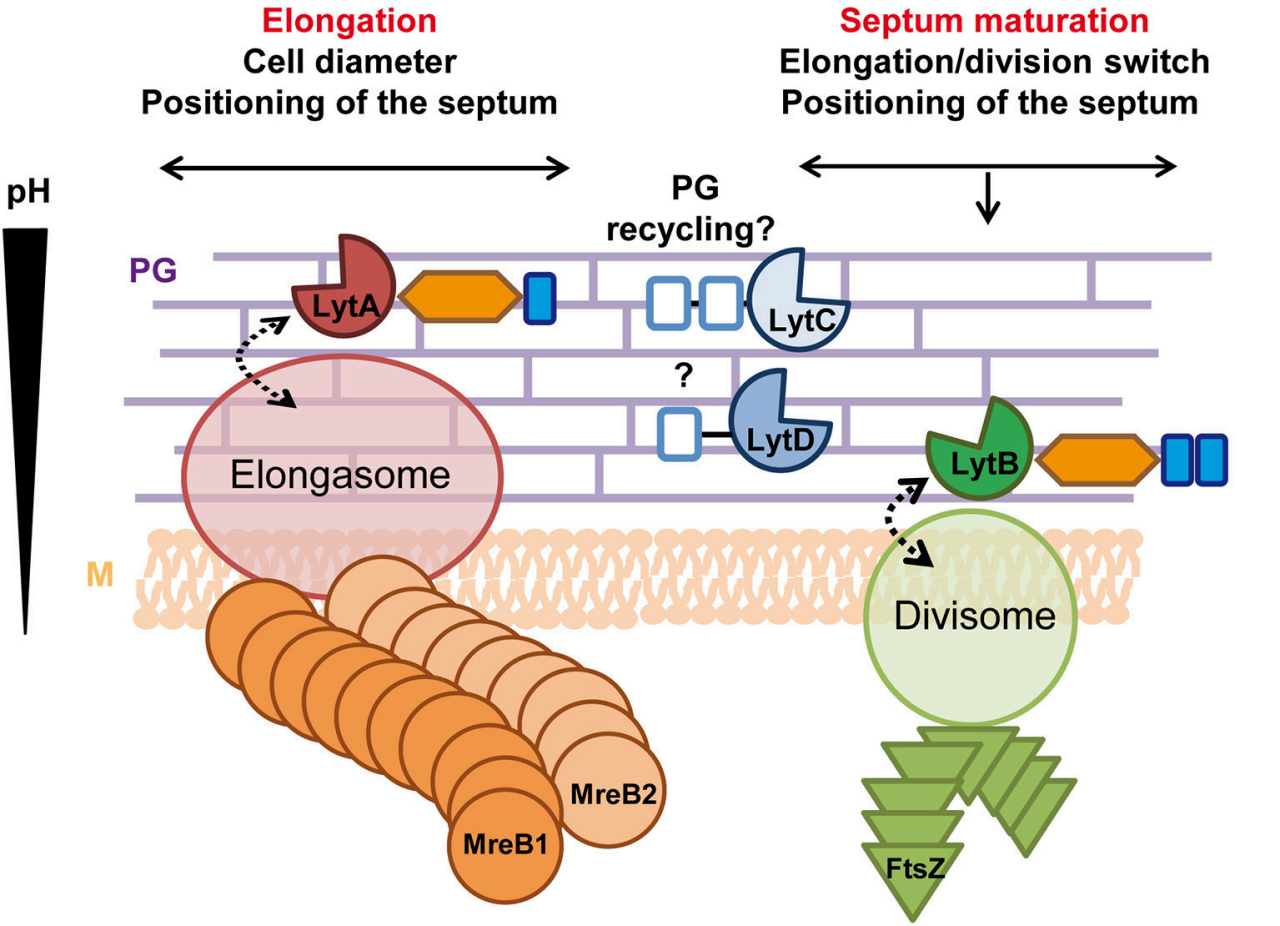
Roles of LytA and LytB in the cell cycle. LytA is mainly involved in cell elongation and in the control of cell diameter. Its absence (probably indirectly) also affects septum positioning. LytA potentially interacts with the elongasome and its basophilic LysM domain suggests that LytA could act in the external PG layers where the pH is higher. LytB is implicated in division (timing, septum maturation, and lateral positioning) and potentially interacts with the divisome. The presence of two acidophilic LysM domains suggests that LytB could be localized close to the cell membrane where the pH is lower. LytC is proposed to be involved in the recycling/catabolism of PG stem peptides while LytD could be an accessory endopeptidase unrelated to the cell cycle. Labeling of accessory domains of Lyt enzymes are as in Figure 1. Dotted arrows indicate potential interactions. PG, peptidoglycan; M, cytoplasmic membrane.

This work shed a new light on our understanding of the roles played by the four NlpC/P60 endopeptidases identified in *L. plantarum*. While LytC and LytD do not appear to be involved in morphogenesis, the paralogous LytA and lytB enzymes play well-separated roles in the cell cycle (Figure 8). We reveal that LytA is a key PGH mainly dedicated to cell elongation while LytB is involved in the division process. Notably, the synthetic co-inhibition of these two processes showed that they are both important for *L. plantarum* growth.

### LytC and LytD Are Not Implicated in Morphogenesis During Standard Growth Conditions

Mutant strains deficient for LytC and/or LytD did not show any observable defect in growth and cell morphology under the tested conditions (Supplementary Figure 1). LytC and LytD display a similar structural organization with accessory SH3b PG-binding domains (Figure 1B). Interestingly, LytC is predicted to be a member of the YkfC subfamily of NlpC/P60 D,L-endopeptidases that only hydrolyze the γ-D-Glu-*meso*-DAP bond of free stem peptides ending with L-Ala (tri, tetra and pentapeptides) (Xu et al., 2015). This activity suggests that members of this subfamily are either involved in the recycling of stem peptide fragments for PG biosynthesis or for their catabolism under nutrient deprivation (Schmidt et al., 2001; Xu et al., 2015). While the physiological role of YkfC remains unknown in *B. subtilis*, the expression of its encoded gene seemed to be regulated and specifically induced in late vegetative growth phase (Smith et al., 2000). Similarly, the *lytC* gene was reported to be upregulated by stress conditions (i.e., T° and NaCl) while the other *lyt* genes were stress-unresponsive (Rolain et al., 2012). In addition, in standard growth conditions, proteomic studies of surface proteins of *L. plantarum* WCFS1 did not reveal the presence of LytC while LytA, LytB and LytD were shown to be produced (Fredriksen et al., 2013). These observations suggest that LytC may play a physiological role in very specific conditions that remain to be determined and could explain the absence of phenotypes regarding growth and morphogenesis in our study.

Intriguingly, the NlpC/P60 domains of LytD and CwlT are closer compared to any other NlpC/P60 domain of *B. subtilis* D,L-endopeptidases (Supplementary Figure 2B). CwlT is not involved in the cell cycle as it is involved in the biogenesis of the DNA transfer machinery required for the conjugation of ICE*Bs1* (Fukushima et al., 2007). The similarity of LytD with CwlT may indicate its involvement in an accessory process such as predation (e.g., fratricins) or the trans-envelope assembly of a secretion/uptake apparatus as previously reported for some members of the NlpC/P60 family (Xu et al., 2015). It is noteworthy that a range of streptococcal fratricins that are specifically induced during competence development are modular enzymes including SH3 domains and closely-related NlpC/P60 domains (Berg et al., 2012). Based on these *in silico* analyses, additional investigations are thus needed to determine the exact function of LytC and LytD.

### LytA Is a Major NlpC/P60 Endopeptidase Involved in the Control of Cell Length

Conditional inactivation of *lytA* and time-lapse experiments revealed its major role in the cell elongation pathway of *L. plantarum* (Figure 4). The absence of LytA resulted in elongation arrest together with misplaced septa, leading to unseparated round cells of various sizes (Figures 3, 4, Supplementary Figures 4, 5). Interestingly, complementation studies of the conditional *lytA* mutant with LytA variants carrying the LytB NlpC/P60 domain, or deleted of either the LysM domain or the AST domain, showed a recovery of elongation without a restoration of cell width, which indicates an additional implication of LytA in the control of the cell diameter (Supplementary Figures 11, 12). Moreover, these dramatic alterations of cell shape were more probably due to a lack of the LytA endopeptidase activity, as shown by complementation studies with a LytA catalytic inactive mutant (Figure 2). In addition, alterations of division planes and cell aggregation do not seem to be the consequence of a lack of elongation as the inactivation of MreBCD, a well-known player of elongation in other rod-shaped Gram-positive bacteria only leads to the formation of well-defined round cells in *L. plantarum* (Supplementary Figure 6). In *B. subtilis*, which displays a similar composition of the stem peptide and the same cross-linking (Vollmer et al., 2008a), CwlO and LytE were shown to, respectively control the cell longitudinal axis and cell diameter, being altogether required for the elongation process (Hashimoto et al., 2012; Domínguez-Cuevas et al., 2013; Meisner et al., 2013). Thus, the LytA enzyme of *L. plantarum* seems to recapitulate the combined activity of CwlO and LytE, which might be consistent with a lower level of PGH redundancy in *L. plantarum* (Smith et al., 2000; Rolain et al., 2012). However, an intriguing consequence of the absence of LytA activity is the misplacement of septa (Supplementary Figures 4, 5) and a lack of a clear localization of FtsZ in round cells (Figure 3). Apart from the Min system (Bernard et al., 2012), no positive or negative players regulating the positioning of the divisome have been identified in *L. plantarum*. Our analysis of PG composition suggests that LytA is responsible of the cleavage of most γ-D-Glu-*meso*-DAP bonds. As products of this hydrolysis represent around 10% of total disaccharides-peptides in the WT profile of muropeptides (Supplementary Table 2), this could indicate that LytA plays an important role in PG remodeling that could indirectly affect either the Min system or unknown players involved in the positioning of the division plane.

### LytB Is Involved in Septum Maturation and Timing of Division

Cell measurements, positioning of septa, and time-lapse experiments showed that LytB is a key player in the regulation of cell division in *L. plantarum* (Figure 8). Indeed, LytB-deficient cells displayed heterogeneous lengths with misplaced division sites along the longitudinal cell axis (Figures 5, 6, Supplementary Figures 7, 8). Our results also suggest that the maturation of the septum is delayed as septa/Z-rings are present in daughter cells while the maturation of the mid-cell septum is still ongoing (Supplementary Figures 7, 8). This delayed septum maturation could contribute to the altered timing between elongation and division such as observed in time-lapse experiments (Figure 6). Moreover, the positioning of septa in daughter cells is laterally affected and seems desynchronized as bi-septal long cells were observed (see Supplementary Figures 7, 8). In *B. subtilis*, the individual inactivation of LytF, CwlS or LytE, yields filaments of unseparated cells (Fukushima et al., 2006), a phenotype that is reminiscent of that reported for the LytB inactivation in *L. plantarum*. Similarly, the inactivation of NlpC/P60 endopeptidases of *L. casei* BL23 (Lc-P75) and *L. rhamnosus* GG (P75/Msp1) lead to cell separation defects and cell chaining (Claes et al., 2012; Regulski et al., 2012). However, the cell-cycle alterations observed for LytB inactivation seem more complex than a mere cell separation defect. By comparison with LytA inactivation, the absence of LytB did not reveal any alteration in the muropeptide profile compared to the WT (data not shown), which is compatible with a specific role of LytB in PG remodeling (e.g., septum maturation). In addition, we cannot exclude that LytB is intimately associated to the division machinery with a structural role. This could indirectly affect the synchronization and placement of division planes in daughter cells. Alternatively, a local remodeling of PG architecture by LytB could provide a specific signature important for the anchoring of early players and the correct placement of the division machinery. Intriguingly, cell-cycle alterations due to LytB depletion are reminiscent of those previously reported for the MurNAc *O*-acetyl-transferase mutant of *L. plantarum* (OatA^−^) (Bernard et al., 2011a, 2012). As for LytB, the absence of OatA led to an altered timing between division and elongation while OatA overproduction resulted in misplaced septa (Bernard et al., 2012). This suggests that OatA and LytB might contribute to the same process. It is tempting to speculate that PG *O*-acetylation might locally disturb PG hydrolysis, for example, by affecting LysM-mediated binding of LytB to its substrate. Local PG *O*-acetylation and PG hydrolysis at mid-cell could in turn act as a cue for the initiation of cytokinesis.

### Co-inactivation of LytA and LytB Leads to a Synthetic Growth Defect

Interestingly, co-inactivation of LytA and LytB severely affects growth, which might indicate a synthetic lethality (Figure 7). Time-lapse experiments of LytA depletion in the LytB-deficient strain clearly showed that LytA and LytB are involved in two separate processes (Supplementary Figure 10) since the cell morphology defect results in an evolving mixed phenotype with a dominancy of LytA over LytB regarding cell shape alteration. In addition to a lack of cell elongation due to LytA deficiency, LytA is also involved in the correct positioning of the septum in round cells. Consequently, the combination of a second defect in the division process due to LytB inactivation (i.e., delayed septum maturation and altered selection of the division site) may thus explain the synthetic effect of a double mutation. It is well-established that rod-shaped cells can tolerate a defect in cell elongation but are much more sensitive to alterations in cell division (Szwedziak and Löwe, 2013). This severe growth defect is also reminiscent of the co-lethality observed for the co-inactivation of LytE and CwlO in *B. subtilis* (Hashimoto et al., 2012). In this case, while both LytE and CwlO are required for cell elongation, LytE is also involved in cell separation (Domínguez-Cuevas et al., 2013). Thus, the synthetic effect of LytA-LytB deficiency on growth in *L. plantarum* reinforces the essential role played by D,L-endopeptidases of the NlpC/P60 family during the cell cycle of rod-shaped Gram-positive bacteria.

### How Are LytA and LytB Controlled?

*Bacillus subtilis* contains three actin-like cytoskeleton proteins (i.e., MreB, MreBH, and Mbl) that are involved in the control of cell elongation. The activity of the LysM-containing enzyme LytE was proposed to be guided by MreBH (and possibly MreB) while the activity of the coiled-coil domain-containing enzyme CwlO was proposed to be associated to Mbl (Domínguez-Cuevas et al., 2013; Meisner et al., 2013). In addition, CwlO was shown to be under the control of the ABC transporter FtsEX, the latter being required for cell elongation in *B. subtilis* as opposed to cell division in *E. coli* (Yang et al., 2011; Domínguez-Cuevas et al., 2013; Meisner et al., 2013). PGHs (e.g., PcsB of *Streptococcus pneumoniae*) or PGH adaptor proteins (e.g., EnvC in *E. coli*) that are controlled by the FtsEX complex contain at least one coiled-coil domain that directly interacts with the surface-exposed FtsX protein (Sham et al., 2011; Yang et al., 2011). A similar interaction has been proposed between CwlO and FtsX (Domínguez-Cuevas et al., 2013). Intriguingly, none of the 12 putative PGHs of *L. plantarum* contains predicted coiled-coil domain (COILS prediction tool, https://toolkit.tuebingen.mpg.de/#/tools/pcoils). Moreover, no canonical FtsEX could be identified in the *L. plantarum* genome based on a search of conserved domains COG2884 and COG2177 for FtsE and FtsX, respectively. This suggests that LytA and/or LytB might be controlled by another mechanism or different players.

In this work, we also performed complementation experiments of the *lytA* mutant with chimeric proteins (i.e., LytA-NlpC/P60_LyB_ and LytB-NlpC/P60_LytA_). The results showed that the accessory domains predominates over the catalytic domain to confer their specific function to the Lyt enzymes (Supplementary Figure 11). In various LysM-containing PGHs, it has been shown that deletion of LysM domain(s) leads to less active or even inactive enzymes (Steen et al., 2005; Layec et al., 2009; Frankel and Schneewind, 2012). In addition, it was observed that the number of LysM domains per PGH (cooperative binding) and their IPs play a major role in their PG-binding capacity (Visweswaran et al., 2011). The presence of one LysM domain with a basic IP in LytA compared to two LysM domains with acidic IPs in LytB may indicate that the LysM domain of LytA have a lower PG-binding capacity in vicinity of the cell membrane, which is more acidic compared to external PG layers (Jolliffe et al., 1981). In this case, LytA would be able to bind PG and be more active in external layers of the cell wall, where PG is more stretched and its hydrolysis required for cell elongation (Figure 8; Lee and Huang, 2013). Conversely, the presence of acidophilic LysM domains in LytB suggests that it could be active in close proximity with the cell membrane, which would be compatible with its role in the maturation and the positioning of the septum.

The roles of the glycosylated AST domains of LytA and LytB remains unexplored. In the Acm2 glucosaminidase of *L. plantarum*, the AST domain and more specifically its glycosyl residues were shown to negatively impact on the enzymatic activity (Rolain et al., 2013). The purification of glycosylated and non-glycosylated variants of LytA and LytB was attempted in different hosts but were unsuccessful so far (data not shown). Besides a direct impact on enzymatic activity, AST-related PGH domains were hypothesized to be involved in subcellular targeting (Huard et al., 2003; Eckert et al., 2006; Claes et al., 2012; Lebeer et al., 2012; Regulski et al., 2012). For instance, the AST domains of LytA or LytB and their glycolytic decorations might interact with other cell-cycle proteins in order to associate LytA and/or LytB to specific complexes of cell wall biosynthesis.

## Author Contributions

PH, BH, M-PC-C, and YD conceived and designed the study. M-CD, TR, PC, and AK carried out the laboratory work. M-CD, PC, PH, BH, M-PC-C, and YD analyzed the data. M-CD, PH, and BH wrote the manuscript. All authors read and approved the final manuscript.

### Conflict of Interest Statement

The authors declare that the research was conducted in the absence of any commercial or financial relationships that could be construed as a potential conflict of interest.

## Supplementary Material

Supplementary Text Construction of strains and plasmids.

Supplementary Figure 1 Structural prediction and phylogenic relationships of NlpC/P60 catalytic domains.

Supplementary Figure 2 Impact of LytC and LytD depletion on cell morphology and growth.

Supplementary Figure 3 RP-HPLC separation of muropeptides from *L. plantarum* WT and P_*nisA*_-*lytA* mutant (without, N0; and with nisin, N25).

Supplementary Figure 4 Effect of LytA depletion on septum misplacement in dividing cells.

Supplementary Figure 5 Effect of LytA depletion on septum misplacement in cell aggregates.

Supplementary Figure 6 Cell cycle of the MreB1CD-deficient strain.

Supplementary Figure 7 Effect of LytB inactivation on septum misplacement in dividing long cells.

Supplementary Figure 8 Effect of LytB inactivation on Z rings positioning.

Supplementary Figure 9 Time-lapse microscopy of Δ*lytB* mutant cells showing lysis of small non-growing cells.

Supplementary Figure 10 Cell cycle of the double LytA LytB deficient strain.

Supplementary Figure 11 Importance of accessory LytA domains for its morphogenic function.

Supplementary Figure 12 Effect of the individual deletion of LytA domains on cell morphology.

Supplementary Table 1 Primers used in this study.

Supplementary Table 2 Disaccharide (Ds)-peptide composition of PG from *L. plantarum* WT and P_*nisA*_-*lytA* mutant (without, N0; and with nisin, N25).

Supplementary Movie 1 Cell cycle of the WT (NZ7100).

Supplementary Movie 2 Cell cycle of the conditional *lytA* mutant (MCD202, N0).

Supplementary Movie 3 Cell cycle of the conditional *mreB1CD* mutant (MCD203, N0).

Supplementary Movie 4 Cell cycle of the Δ*lytB* mutant (TR0015), Cell 1.

Supplementary Movie 5 Cell cycle of the Δ*lytB* mutant (TR0015), Cell 2.

Supplementary Movie 6 Cell cycle of the double LytA-LytB deficient strain (MCD20215, N0), Cell 1.

Supplementary Movie 7 Cell cycle of the double LytA-LytB deficient strain (MCD20215, N0), Cell 2.
