# Supplementary material for: Distinct and Specific Role of NlpC/P60 Endopeptidases LytA and LytB in Cell Elongation and Division of Lactobacillus plantarum
Source: Front Microbiol. 2019 Apr 12;10:713. doi: 10.3389/fmicb.2019.00713 (PMC6473061; doi:10.3389/fmicb.2019.00713)
Supplement: Supplementary Text — Construction of strains and plasmids. [file Data_Sheet_1.PDF]

Supplementary Material

1 Supplementary Text

Construction of strains and plasmids

Construction of conditional and disruption mutants.

Suicide plasmids used to construct *lytA*, *lytC*, *mreB1CD* conditional mutants were assembled as follows. Initially, constructions were performed in plasmid pGIM008 (Palumbo et al., 2006), which contains the *nisA* promoter (P_{nisA}). To avoid transcriptional interference from upstream promoters, a strong transcriptional terminator was cloned upstream of P_{nisA}. For this purpose, the terminator of the *ldhL* gene (T_{ldhL}) was PCR-amplified from the chromosome of NZ7100 with primers MC_Tldh_pGIM008_1 and MC_Tldh_pGIM008_2. Plasmid pGIM008 and the amplicon were digested with *Bgl*II and ligated together, resulting in plasmid pGIMCD700 (T_{ldhL}-P_{nisA}). Then, 3'-end truncated fragments of *lytA, lytC,* and *mreB1* CDS (*lytA'*, *lytC'*, and *mreB1'*, respectively) were cloned in translation fusion (ATG fusion) with the P_{nisA} expression cassette. The *lytA'*, *lytC'*, and *mreB1'* inserts were PCR-amplified and flanked of restriction sites (*Nco*I and *Pst*I, *Pst*I and *Sac*I, and *Nco*I and *Pvu*I, respectively) with primer pairs MC_pJIM_lytA_1 and MC_pGIM_lytA_PstI, MC_pGIM_lytC_PstI and MC_pGIM_lytC_SacI, and MC_MreB1_NcoI and MC_mreB1_PvuI, respectively. PCR products were digested and cloned in their respective restriction sites of plasmid pGIMCD700 to generate the intermediate plasmids pGIMCD702 (T_{ldhL}-P_{nisA}-*lytA'*), pGIMCD706 (T_{ldhL}-P_{nisA}-*lytC'*), and pGIMCD703 (T_{ldhL}-P_{nisA}-*mreB1'*). The T_{ldhL}-P_{nisA}-*lytA'*, T_{ldhL}-P_{nisA}-*lytC'*, and T_{ldhL}-P_{nisA}-*mreB1'* cassettes were PCR-amplified and flanked of *Nde*I and *Hin*dIII restriction sites with primers pUC_Cm_V2_1 and pUC_Cm_V2_2. Digested amplicons were cloned in the same restriction sites of plasmid pUC18Cm to generate the final suicide vectors pGIMCD202 (T_{ldhL}-P_{nisA}-*lytA'*), pGIMCD206 (T_{ldhL}-P_{nisA}-*lytC'*) and pGIMCD203 (T_{ldhL}-P_{nisA}-*mreB1'*). These plasmids were used to construct the conditional mutant strains MCD202 (P_{nisA}-*lytA*), MCD206 (P_{nisA}-*lytC*), and MCD203 (P_{nisA}-*mreB1CD*).

The suicide plasmid used to construct the *lytC* disruption mutant was assembled as follows. The internal *lytC* fragment (*'lytC'*) was PCR-amplified and flanked of restriction sites *Pst*I and *Hin*dIII with primers MC_lytC_KOSCO_1 and MC_lytC_KOSCO_2. The pUC18Cm and the insert were digested by *Pst*I and *Hin*dIII and ligated together to obtain the disruption vector pGIMCD208. This suicide plasmid was used to generate the mutant strain MCD208 (*lytC*::pGIMCD208).

Construction and validation of the ComS-inducible expression plasmid.

The low-copy vector containing the ComS-inducible expression system was constructed as follows. We started from plasmid pSIP103-104 (Desguin et al., 2015) containing the reporter gene *gusA*. The insert *comR*-P_{comS} from *Streptococcus thermophilus* LMD-9 was PCR-amplified and flanked of restriction sites *Sal*I and *Nco*I with primers MC_ComRS_1 and MC_ComRS_2, respectively. The pSIP103-104 and the insert were digested with the corresponding enzymes and ligated together to obtain pGIMCD101 (*comR*-P_{comS}-*gusA*). The T_{ldhL} was PCR-amplified and flanked of restriction sites

*Sac*I and *Nco*I with primers MC_termi_LDH_1 and MC_termi_LDH_2, respectively. The pGIMCD101 was amplified by inverted PCR with primers MC_SacIComS_1 and MC_SacIComS_2. This amplicon was self-ligated to obtain an intermediate vector without P_{comS} and with a new *Sac*I restriction site. This intermediate vector and the T_{ldhL} amplicon were digested with *Sac*I and *Nco*I and ligated together to generate the promoter-probe vector pGIMCD102 (*comR*-T_{ldhL}-*'gusA*). The ComS-regulated promoter $P_{shp0064}$ from *S. thermophilus* LMD-9 (Fontaine et al., 2013) was PCR-amplified and flanked of restriction sites *Swa*I and *Nco*I with primers MC_Pshp0064_1 and MC_Pshp0064_2, respectively. The obtained insert and pGIMCD102 were digested by *Swa*I and *Nco*I and ligated to obtain pGIMCD106 (*comR*-T_{ldhL}-$P_{shp0064}$-*gusA*). The functionality of the ComRS system was validated in strain NZ7100(pGIMCD106) by measuring the β-glucuronidase (GUS) activity (Desguin et al., 2015) in response to increasing concentrations of ComS (0, 2, 4, 10 µM). For this purpose, bacteria were inoculated at an OD_{600} of 0.1, grown in MRS containing erythromycin with or without ComS, and collected after 1h30 (OD_{600} of 0.3) for GUS activity assays. The specific GUS activity (Arbitrary Unit, A.U.) is defined as the amount (µmol) of *p*-nitrophenyl-β-d-glucuronide hydrolyzed per min and per mg total proteins. At ComS concentrations of 0, 2, 4, and 10 µM, measured GUS activities were 5.3, 152.4, 370.9, and 435.4 A.U., respectively. To create a multiple cloning site and to delete the *gusA* gene, plasmid pGIMCD106 was amplified by reverse PCR with primers MC_Pshp0064_2 and MC_TpepN_XbaI and self-ligated, resulting in the final expression vector pGIMCD107 (*comR*-T_{ldhL}-$P_{shp0064}$-MCS).

Construction and validation of complementation strains.

The complementation vectors expressing *lytA*, *lytB* and *lytA** were obtained as follows. The *lytA* and *lytB* CDSs were PCR-amplified from the chromosome of NZ7100 with primer pairs MC_pSIP_lytA_1 and MC_pSIP_lytA_2, and MC_pSIP_lytB_1 and MC_pSIP_lytB_2, respectively. Inserts were flanked of restrictions sites *Nco*I and *Eco*RI. The expression plasmid pGIMCD107 was digested by the corresponding restriction enzymes and ligated with the inserts in order to obtain complementation vectors pGIMCD117 ($P_{shp0064}$-*lytA*) and pGIMCD118 ($P_{shp0064}$-*lytB*). To generate a catalytic mutant of LytA (LytA*, Cys_{284} to Ala), pGIMCD117 was amplified by inverse PCR with primers MC_lytA_mutcat_1 and MC_lytA_mutcat_2. The PCR product was phosphorylated and ligated to obtain pGIMCD121 ($P_{shp0064}$-*lytA**). The presence of the mutation was checked by sequencing using primers MC_pSIP_fluo_verif_1 and MC_pSIP_fluo_verif_2. The respective production of LytA, LytA*and LytB from complementation strains MCD202(pGIMCD117), MCD202(pGIMCD121), and TR0015(pGIMCD118) in presence of ComS (8 µM) was validated by mass spectrometry. For this purpose, the cell pellet was washed once with PBS, resuspended in PBS, and vortexed 30 min at 4°C. Bacteria were then harvested and the supernatant was analyzed by mass spectrometry as reported before (Rolain et al., 2013). Specific trypsinized peptides corresponding to the NlpC/P60 catalytic domain of LytA, LytA*and LytB were identified (data not shown).

Construction of expression plasmids for hybrid and truncated proteins.

The plasmid pGIMCD117 ($P_{shp0064}$-*lytA*) was amplified by reverse PCR with primers MC_pSIP_NlpC_deletion_A_1 and MC_pSIP_NlpC_deletion_A_2 to remove the gene fragment coding for the NlpC/P60 domain and to add restriction sites *Bgl*II and *Xba*I. The PCR product was self-ligated after restriction and phosphorylation to obtain pGIMCD125 expressing the truncated protein LytAΔNlpC/P60. The *lytB* fragment coding for its NlpC/P60 domain was PCR-amplified from pGIMCD118 ($P_{shp0064}$-*lytB*) and flanked of restriction sites *Bgl*II and *Xba*I with primers MC_NlpC_B_1 and MC_NlpC_B_2. The *Bgl*II-*Xba*I digested insert was cloned at the respective

restriction sites of pGIMCD125 to generate plasmid pGIMCD126 expressing the hybrid protein LytA-NlpC/P60$_{LytB}$. The pGIMCD118 (P$_{shp0064}$-*lytB*) was amplified by inverse PCR with primers MC_pSIP_NlpC_deletion_A_1 and MC_pSIP_NlpC_deletion_B_2 to remove the *lytB* fragment coding for the NlpC/P60 domain and to add restriction sites *Bgl*II and *Xba*I. The PCR product was self-ligated after phosphorylation to generate pGIMCD132 producing LytBΔNlpC/P60. The *lytA* fragment coding for the NlpC/P60 domain was PCR-amplified and was flanked of restriction sites *Bgl*II and *Xba*I with primers MC_NlpC_A_1 and MC_NlpC_A_2. The pGIMCD132 and the insert were digested with the appropriate enzymes and ligated to generate pGIMCD124 producing the hybrid protein LytB-NlpC/P60$_{LytA}$.

The plasmid pGIMCD122 expressing LytAΔLysM was obtained by inverse PCR amplification of pGIMCD117 (P$_{shp0064}$-*lytA*). Primers MC_pSIP_lysM_deletion_A_1 and MC_pSIP_lysM_deletion_A_2 were used to remove the gene fragment coding for the LysM domain and to add restriction sites *Bgl*II and *Xba*I. The PCR product was self-ligated after digestion and phosphorylation to obtain pGIMCD122 (P$_{shp0064}$-*lytAΔLysM*). The plasmid pGIMCD128 (P$_{shp0064}$-*lytAΔAST*) expressing LytAΔAST was obtained by the same strategy with primers MC_delta_AST_LytA_1 and MC_delta_AST_LytA_2.

Construction of the FtsZ-GFP$^+$ expression plasmid.

The *gfp*$^+$ CDS was PCR-amplified from plasmid pAE03 (Eberhardt et al., 2009) and flanked of restriction sites *Pst*I and *Xba*I with primers MC_GFPpAE03_PstI and MC_GFPpAE03_XbaI, respectively. The plasmid pNZ8048 (Kuipers et al., 1995) and the amplicon were digested by *Pst*I and *Xba*I, and ligated together to obtain pGIMCD301 (P$_{nisA}$-*gfp*$^+$). The P$_{nisA}$-*gfp*$^+$ cassette was PCR-amplified from pGIMCD301 and flanked of *Kpn*I restriction sites with primers MC_K7PnisGFPKpnI_5 and MC_K7PnisGFPKpnI_3. The pGIMCD102 and the cassette were digested with *Kpn*I and ligated to obtain pGIMCD113 (T$_{ldhL}$-P$_{nisA}$-*gfp*$^+$). The *ftsZ* gene was PCR-amplified from the chromosome of NZ7100 and flanked of *Not*I restriction sites with primers MC_pSIP_FtsZ_GFP_1 and MC_pSIP_FtsZ_GFP_2. The pGIMCD113 and the insert were digested by *Not*I and ligated together to obtain pGIMCD116 (T$_{ldhL}$-P$_{nisA}$-*ftsZ*-*gfp*$^+$). The expression plasmid pGIMCD107 was amplified by reverse PCR with primers MC_modif_MCS_1 and MC_modif_MCS_2 to modify the MCS and to obtain pGIMCD115 (*comR*-T$_{ldhL}$-P$_{shp0064}$-MCSbis). The *ftsZ*-*gfp*$^+$ insert was PCR-amplified from pGIMCD116 and flanked of restriction sites *Xba*I and *Xma*I with primers MC_FtsZ_GFP_com_1 and MC_FtsZ_GFP_com_2. The amplicon was then cloned at the same restriction sites in pGIMD115 to obtain the final low-copy vector pGIMCD110 (T$_{ldhL}$-P$_{shp0064}$-*ftsZ*-*gfp*$^+$).

Reference

Desguin, B., Goffin, P., Bakouche, N., Diman, A., Viaene, E., Dandoy, D. et al. (2015). Enantioselective regulation of lactate racemization by LarR in *Lactobacillus plantarum*. *J. Bacteriol.* 197, 219-230. JB.02192-14 [pii];10.1128/JB.02192-14 [doi].

2 Supplementary Figures and Tables

2.1 Supplementary Figures

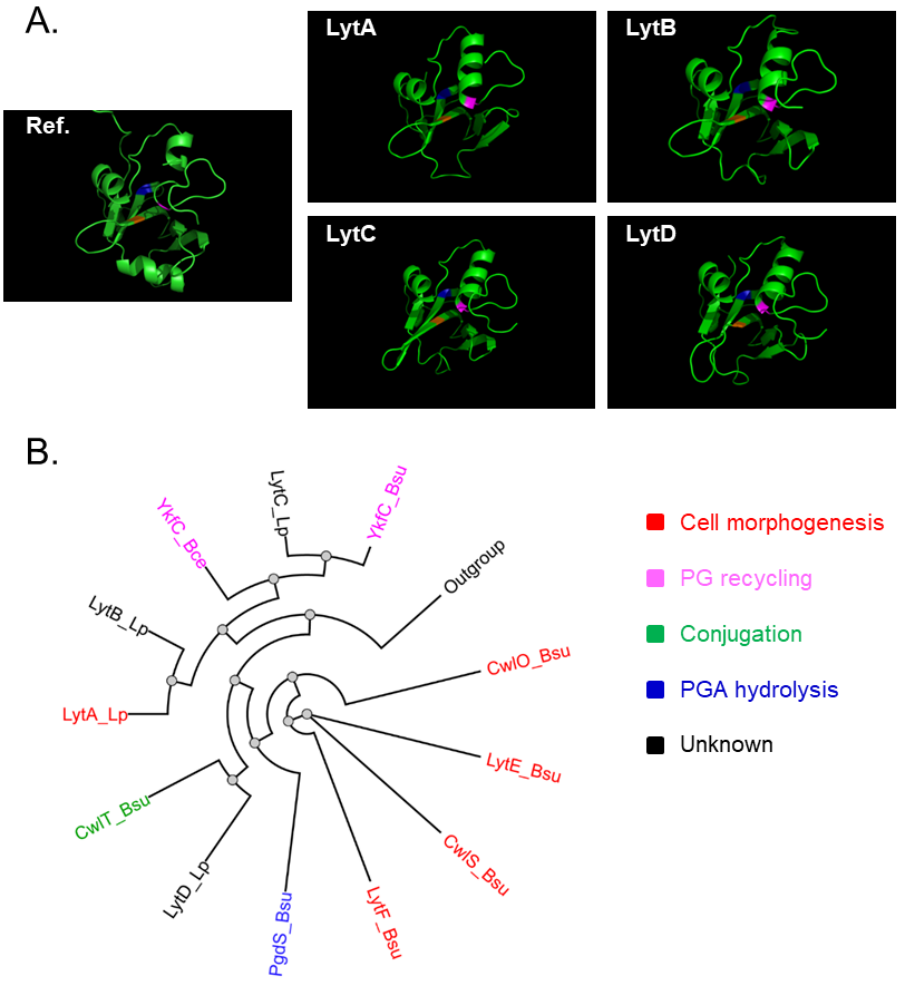

Supplementary Figure 1. Structural prediction and phylogenetic relationships of NlpC/P60 catalytic domains (A) Structural prediction of NlpC/P60 domains of Lyt endopeptidases of *L. plantarum*. LytA, LytB, LytC and LytD structures are based on the alignment with NlpC/P60 npun_r0659 from *Nostoc punctiform* (PDB n° c2fg0B, Reference). Proteins respectively share 36 %, 26 %, 35 % and 29 % of identity with this model. Catalytic residues are coloured: Cys in magenta, His in orange and Asp in blue. Structural predictions were performed with Phyre 2.0 (http://www.sbg.bio.ic.ac.uk/phyre2/). **(B)** Phylogenic relationships between NlpC/P60 domains of D,L-endopeptidases of *B. subtilis* and *L. plantarum*. Protein domain sequences were aligned with Clustal Omega using default parameters (https://www.ebi.ac.uk/Tools/msa/clustalo/). The PHYLIP output file was used to generate the phylogenetic tree (dendrogram presentation for clarity) with PhyML 3.0 (http://www.atgc-montpellier.fr/phyml/) based on the maximum likelihood algorithm. The lysozyme-like domain of CwlT of *B. subtilis* was used as outgroup. The color code corresponds to predicted or validated function of NlpC/P60 before this work. Lp, *L. plantarum*; Bsu, *Bacillus subtilis*, and Bce, *Bacillus cereus*.

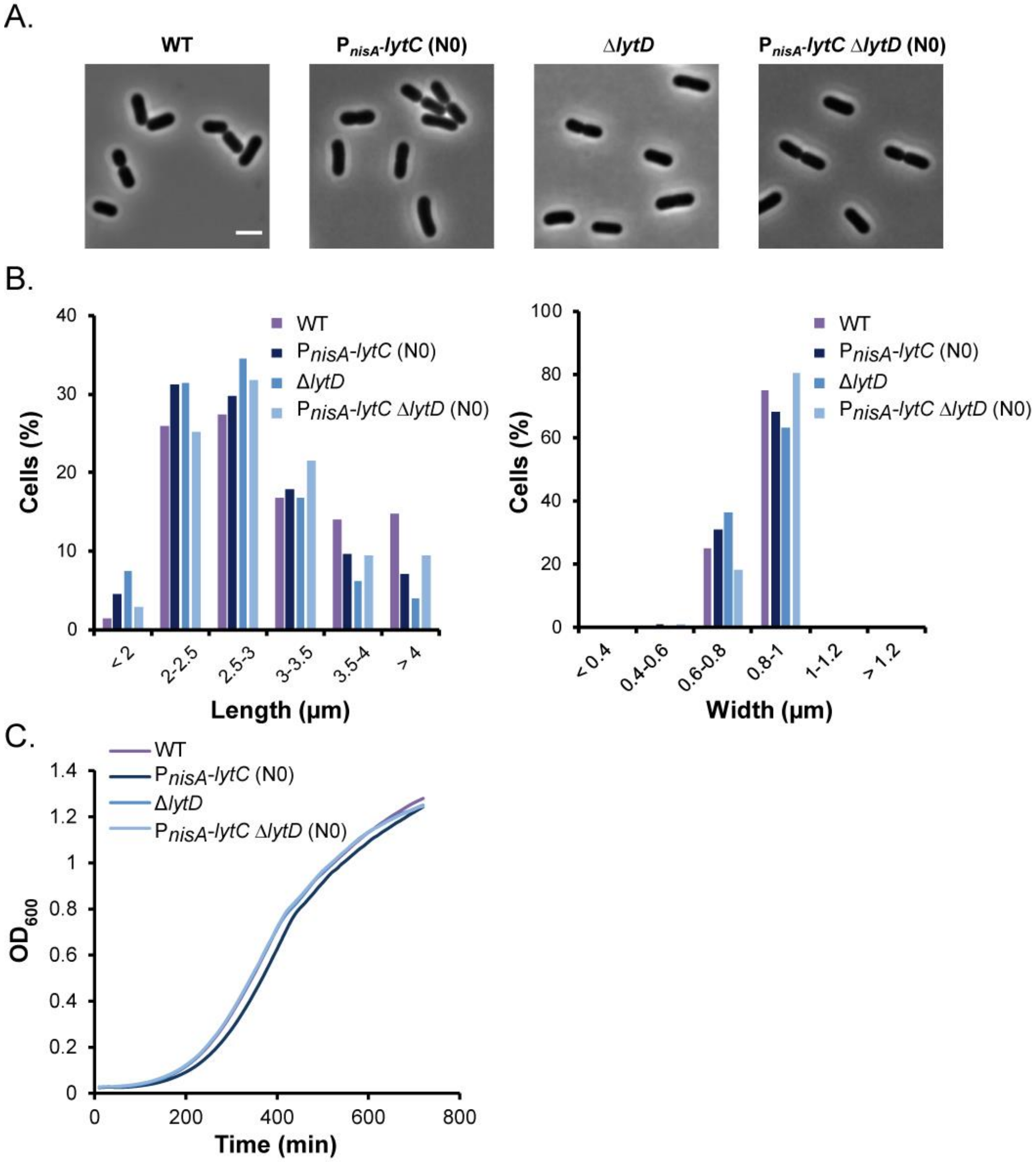

Supplementary Figure 2. Impact of LytC and LytD depletion on cell morphology and growth. (A) Phase contrast (PC) microscopy of WT, P_{nisA}-*lytC* mutant (not induced, N0), Δ*lytD* mutant, and the double P_{nisA}-*lytC* Δ*lytD* mutant (N0). Bacteria were grown in MRS without nisin and with chloramphenicol when required. Observations were performed in exponential phase from at least two independent experiments. Scale bar is 2 µm. **(B)** Length and width of the cell population measured by microbeTracker with *n* > 200 cells (data were obtained from at least two independent experiments). **(C)** Growth curves of WT, P_{nisA}-*lytC* mutant (N0), Δ*lytD* mutant, and P_{nisA}-*lytC* Δ*lytD* mutant (N0). Strains were grown in MRS with chloramphenicol when required. Curves represent the mean value from three measurements.

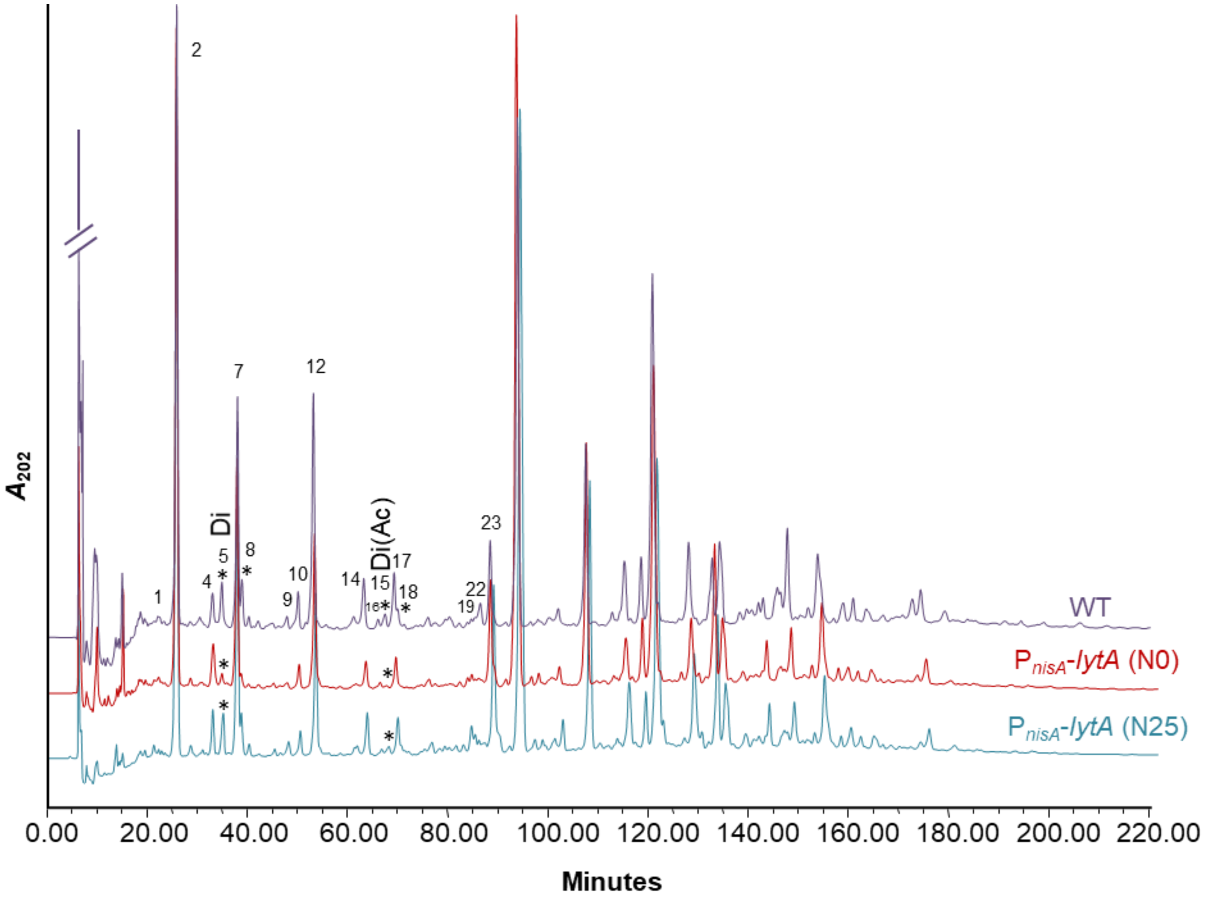

Supplementary Figure 3. RP-HPLC separation of muropeptides from *L. plantarum* WT and P_{nisA}-*lytA* mutant (without, N0; and with nisin, N25). The peak numbers refer to supplementary Table S2. Peaks 5, 8, 15, and 18 (asterisks) are muropeptides resulting from cleavage of a D-iGln-mDAP(NH_2) bond. Di, disaccharide dipeptide (L-Ala-D-iGln) (peak 5); Di(Ac), *O*-acetylated disaccharide dipeptide (L-Ala-D-iGln) (peak 15).

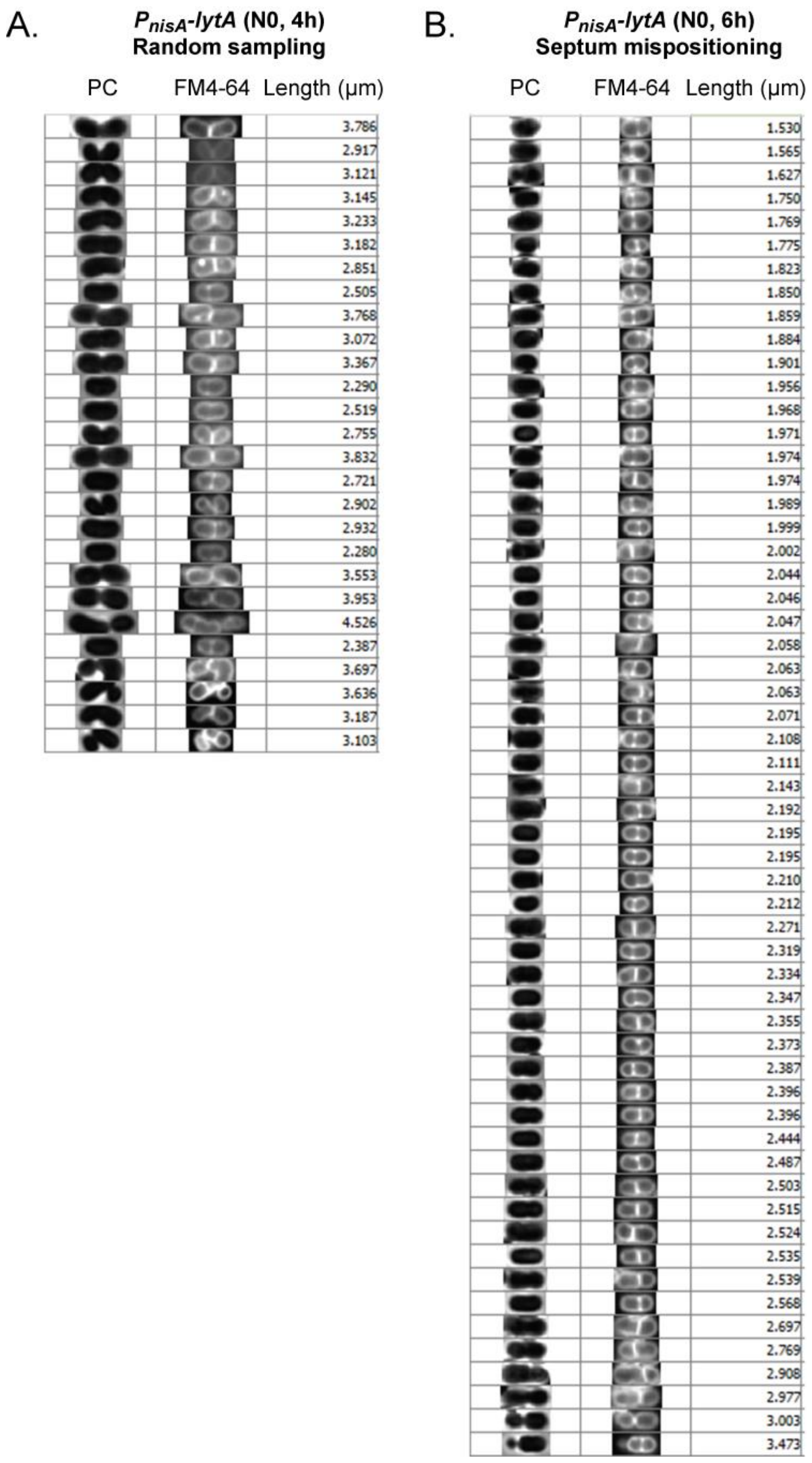

Supplementary Figure 4. Effect of LytA depletion on septum misplacement in dividing cells. (A) Random sampling of P_{nisA}-*lytA* mutant cells observed after 4 hours of nisin depletion by phase contrast (PC) microscopy and FM4-64 staining. **(B)** Selection of dividing cells of P_{nisA}-*lytA* mutant observed after 6 hours of nisin depletion, which display misplaced septa (defects in lateral positioning and orientation). Misplaced septa concerns more than 70 % of dividing cells (total sample of 194 out of 303 examined cells from triplicates). Images and measurements of cell length (µm) were generated with MicrobeJ.

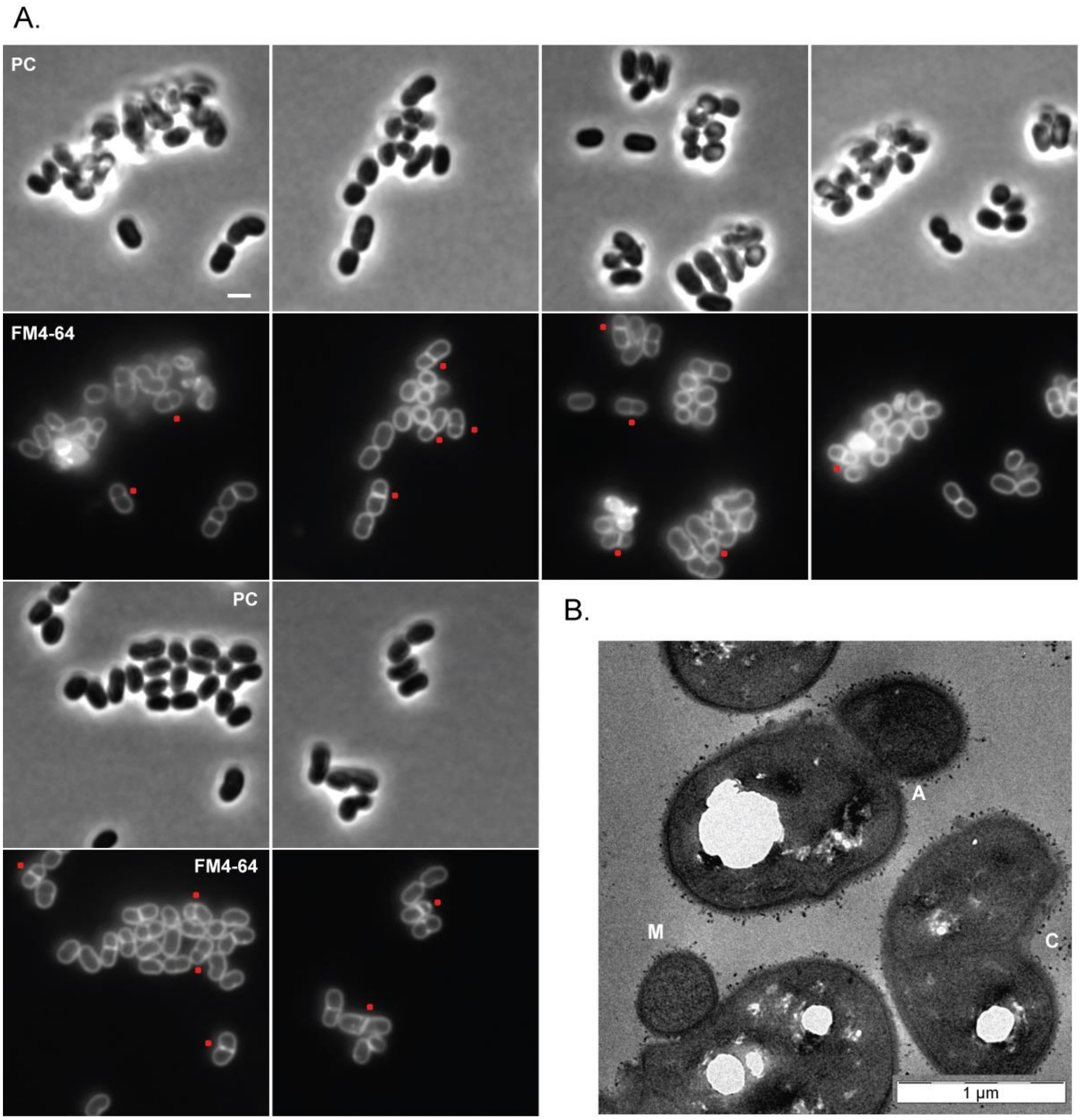

Supplementary Figure 5. Effect of LytA depletion on septum misplacement in cell aggregates. **(A)** Selection of cell aggregates of P$_{nisA}$-*lytA* mutant observed after 6 hours of nisin depletion by phase contrast (PC) microscopy and FM4-64 staining. Red squares indicate cells with misplaced septa. The scale bar is 2 µm. **(B)** Cells of the stable Δ*lytA* mutant observed by transmission electron microscopy (TEM) showing alteration in septum formation/placement (see Rolain et al, 2012 for experimental procedures). A, asymmetrical division; C, curved cell with PG accumulation at division site; and M, minicell.

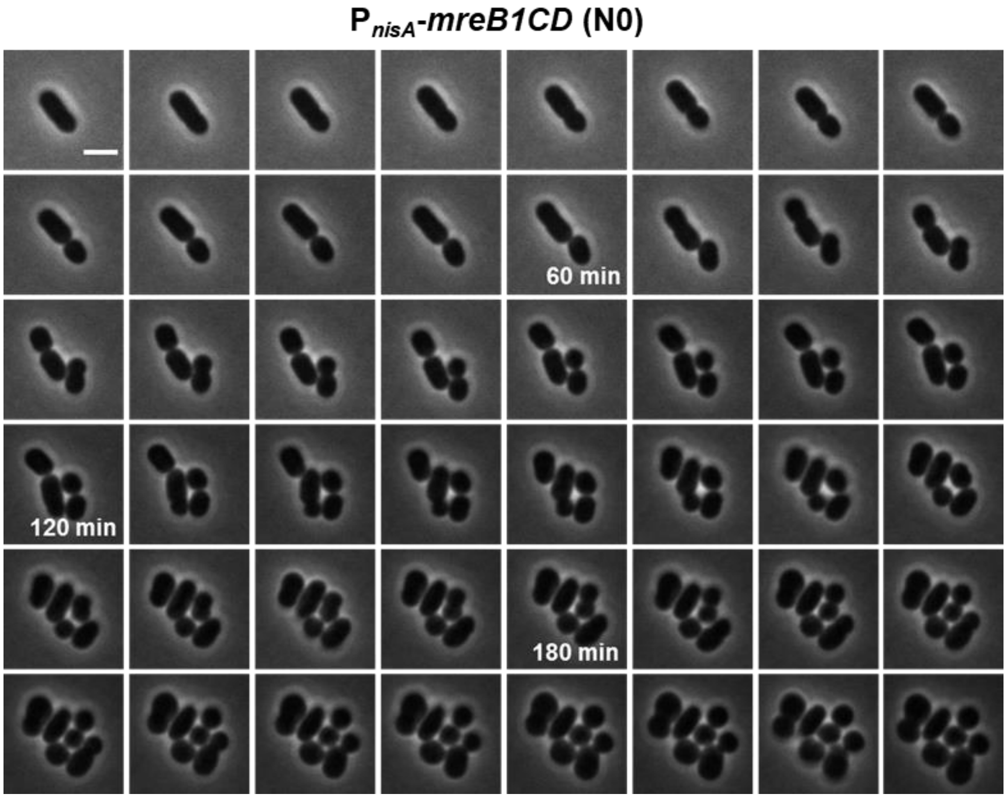

Supplementary Figure 6. Cell cycle of the MreB1CD-deficient strain. Time lapse microscopy (phase contrast) of P_{nisA}-*mreB1CD* mutant cells under nisin depletion. Bacteria were grown on MRS-containing agarose pads at 30°C. The scale bar is 2 µm.

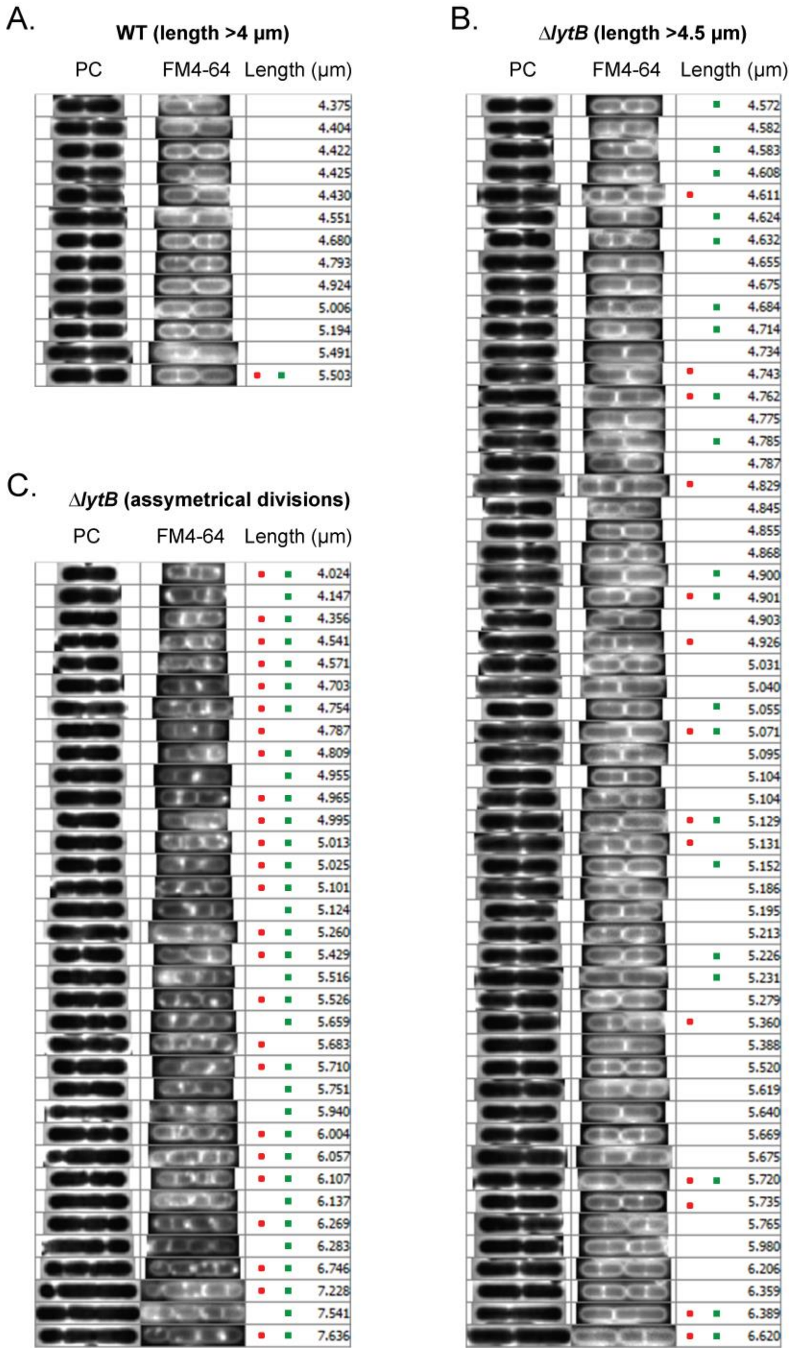

Supplementary Figure 7. Effect of LytB inactivation on septum misplacement in dividing long cells. (A) Random selection of WT cells with a length > 4.0 µm (total sample of 46 out of 1093 examined cells from triplicates). **(B)** Random selection of Δ*lytB* mutant cells with a length > 4.5 µm (total sample of 96 out of 668 examined cells from triplicates). **(C)** Selection of Δ*lytB* mutant cells with a length > 4.0 µm, which display strong lateral misplacement of septa. Red and green squares indicate cells with laterally misplaced septum and desynchronized septum formation in daughter cells, respectively. Cells were collected in exponential growth phase, and observed by phase contrast (PC) microscopy and FM4-64 staining. Images and measurements of cell length (µm) were generated with MicrobeJ.

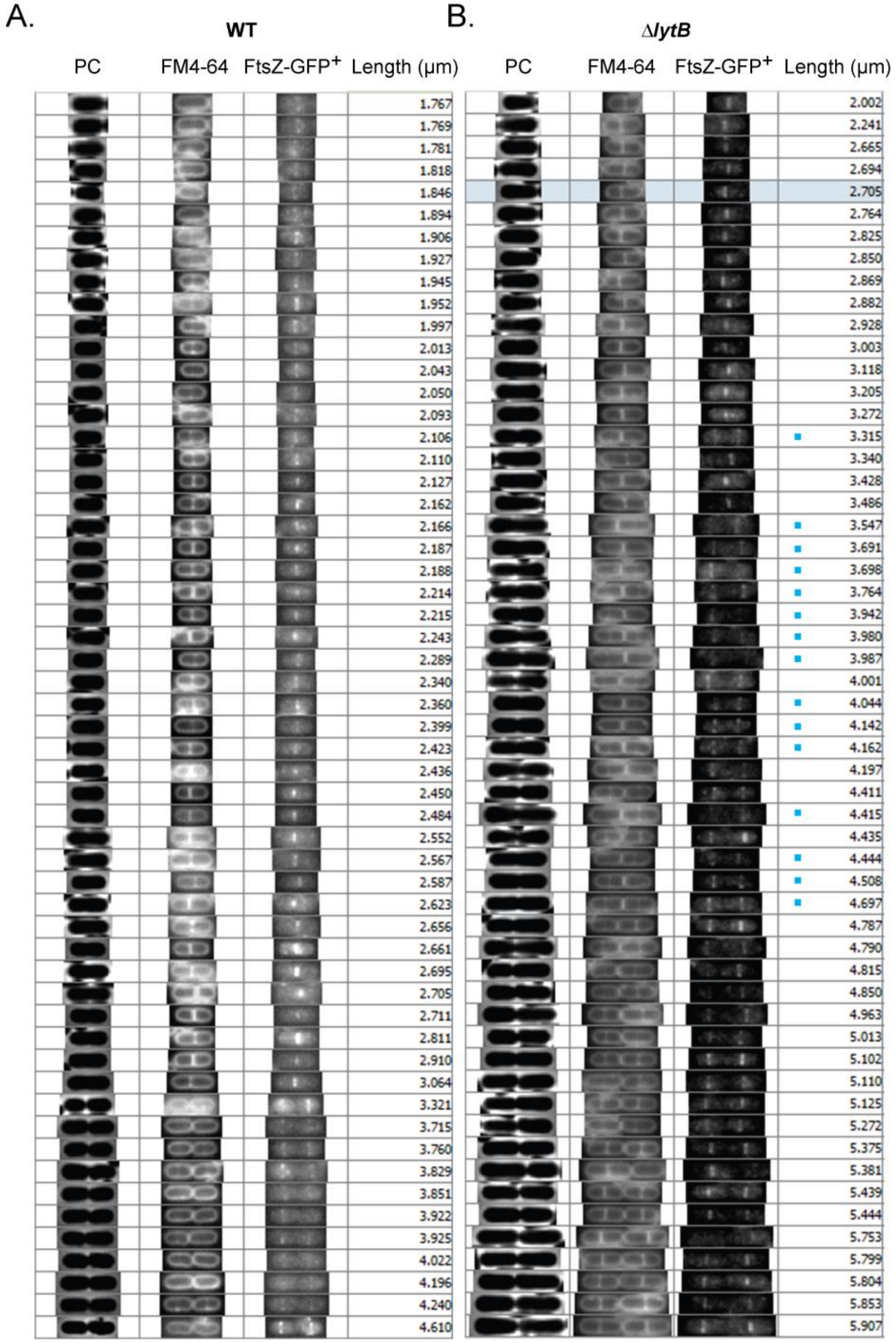

Supplementary Figure 8. Effect of LytB inactivation on Z rings positioning. Selection of fluorescently-labelled WT **(A)** and Δ*lytB* mutant cells **(B)** expressing an FtsZ-GFP$^+$ fusion (P$_{shp0064}$-*ftsZ-fgp*$^+$). Blue squares indicate delayed mid-cell septum maturation with premature migration of Z rings in daughter cells. Bacteria were cultured in MRS with erythromycin and induced with ComS (8 µM, C8). Cultures were moderately shaken after ComS induction. Cells were collected in exponential growth phase, and observed by phase contrast (PC) microscopy and epifluorescence for FM4-64 staining and GFP detection. Cells were selected from triplicates. Images and measurements of cell length (µm) were generated with MicrobeJ.

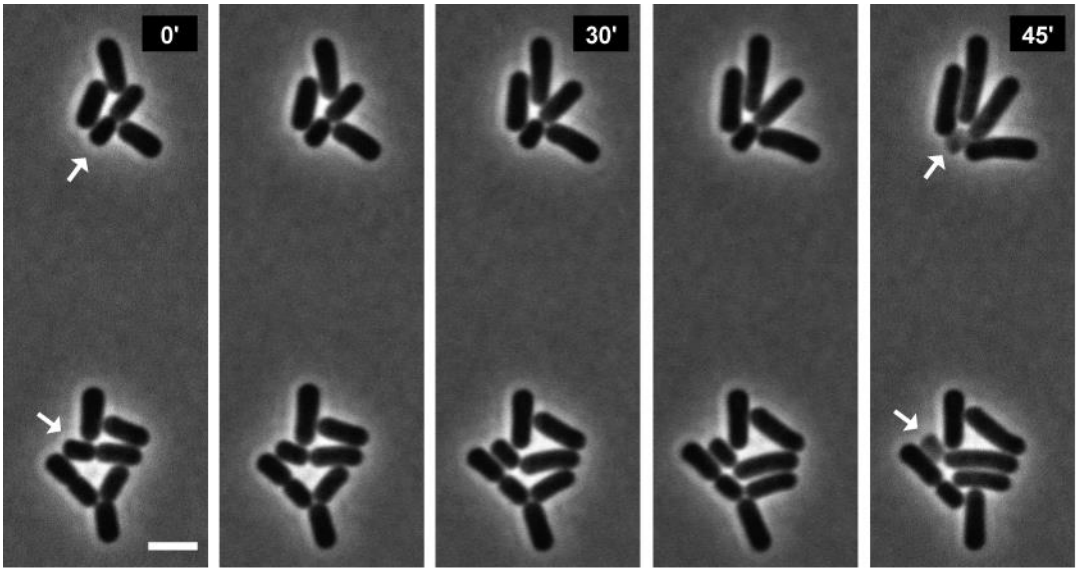

Supplementary Figure 9. Time-lapse microscopy of Δ*lytB* mutant cells showing lysis of small non-growing cells (white arrows). Bacteria were grown on MRS-containing agarose pads at 30°C. The scale bar is 2 µm.

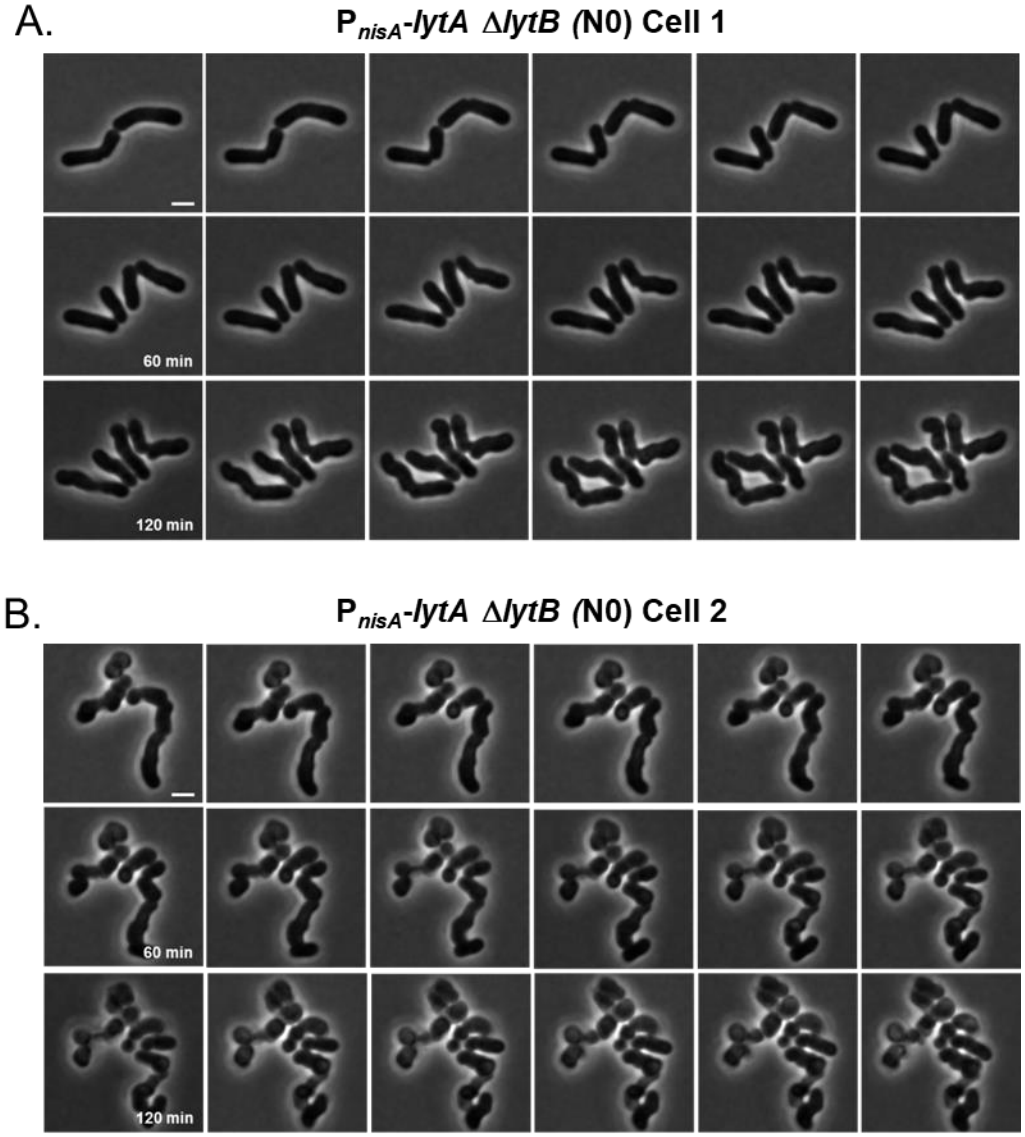

Supplementary Figure 10. Cell cycle of the double LytA LytB deficient strain. Time lapse microscopy (phase contrast) of the P_{nisA}-*lytA* Δ*lytB* mutant cells under nisin depletion. **(A)** and **(B)** P_{nisA}-*lytA* Δ*lytB* mutant cells at different stages of LytA deficiency. Bacteria were grown MRS-containing agarose pads at 30°C. The scale bar is 2 µm.

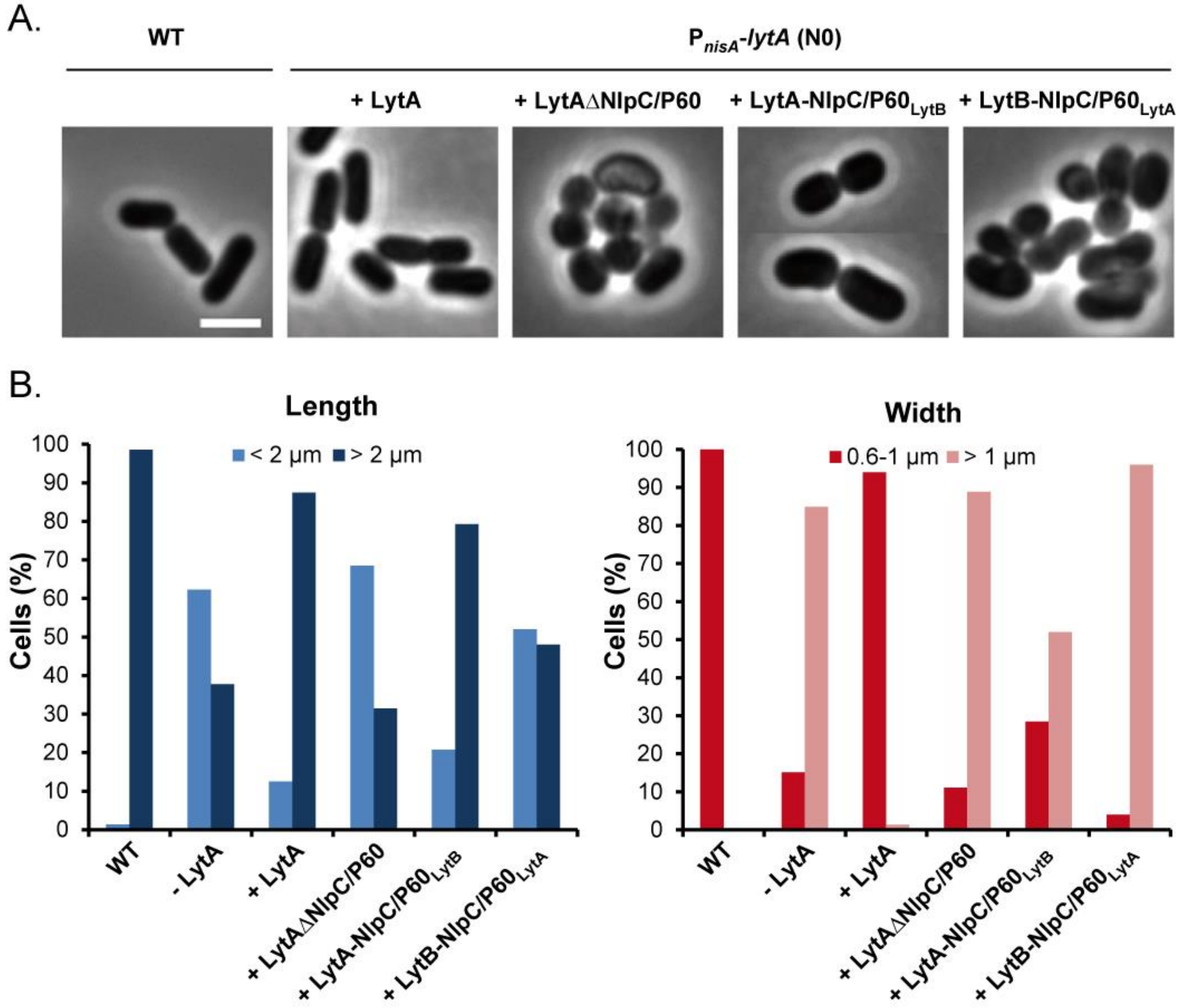

Supplementary Figure 11. Importance of accessory LytA domains for its morphogenic function. (A) Phase-contrast images of cells of WT, P_{nisA}-*lytA* mutant complemented with LytA (+ LytA), P_{nisA}-*lytA* mutant + LytAΔNlpC/P60 (deletion of the catalytic domain), P_{nisA}-*lytA* mutant + LytA-NlpC/$P60_{LytB}$ (LytA accessory domains fused to LytB catalytic domain), and P_{nisA}-*lytA* mutant + LytB-NlpC/$P60_{LytA}$ (LytB accessory domains fused to LytA catalytic domain). Bacteria were grown in MRS without nisin (N0), and in presence of chloramphenicol, erythromycin, and ComS (8 µm, C8) when needed. Cells were collected in mid-exponential growth phase, suspended in PBS, and observed on agarose pads. The scale bar is 2 µm. **(B)** Cell length and width of WT, P_{nisA}-*lytA* mutant complemented with LytA without ComS (- LytA) and with ComS (+ LytA), P_{nisA}-*lytA* mutant + LytAΔNlpC/P60 (N0, C8), P_{nisA}-*lytA* mutant + LytA-NlpC/$P60_{LytB}$ (N0, C8), and P_{nisA}-*lytA* mutant + LytB-NlpC/$P60_{LytA}$ (N0, C8). Frequencies (%) of short (< 2 µm) and long (> 2µm) cells (left) and of cells with small (0.6-1 µm) and large (> 1 µm) width (right) are shown. The P_{nisA}-*lytA* mutant complemented with LytA in presence of ComS (+ LytA) and absence of ComS (- LytA) was used as positive and negative controls, respectively. For (A) and (B), data were obtained from at least two independent experiments. For (B), measures were obtained by using MicrobeTracker with *n* > 50 cells.

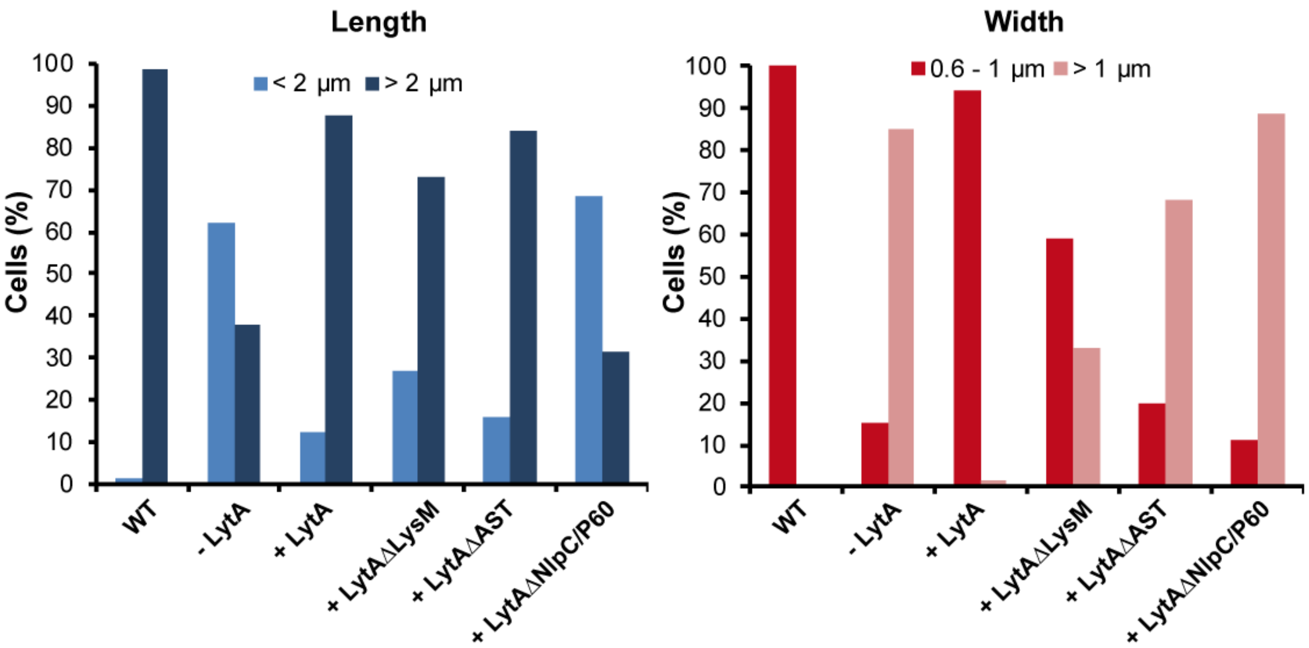

Supplementary Figure 12. Effect of the individual deletion of LytA domains on cell morphology. Cell length and width of WT, P_{nisA}-*lytA* mutant complemented with LytA without ComS (- LytA) and with ComS (+ LytA), P_{nisA}-*lytA* mutant + LytAΔLysM (deletion of the LysM domain; N0, C8), P_{nisA}-*lytA* mutant + LytAΔAST (deletion of the AST domain; N0, C8), P_{nisA}-*lytA* mutant + LytAΔNlpC/P60 (deletion of the catalytic domain; N0, C8). Frequencies (%) of short (< 2 µm) and long (> 2µm) cells (left) and of cells with small (0.6-1 µm) and large (> 1 µm) width (right) are shown. The P_{nisA}-*lytA* mutant complemented with LytA in presence of ComS (+ LytA) and absence of ComS (- LytA) was used as positive and negative controls, respectively. Data were obtained from at least two independent experiments. Measures were generated by using MicrobeTracker with $n > 50$ cells.

2.2 Supplementary Tables

Supplementary Table 1. Primers used in this study

Primer	Sequence (5' to 3')	Target
**pSIP409 derivatives**		
**Construction**		
MC_ComRS_1	TCGAGTCGACAACAATATGCAGTTCTGA	*comR*-P$_{comS}$ from LMD-9
MC_ComRS_2	CATGCCATGGTATAACTCCTTTTAAC	
MC_SacIComS_1	TTTCTATAAACCATCTGCCAATTTTCC	pGIMCD101
MC_SacIComS_2	ATCGAGCTCGGAGTTATACCATGGTACG TCC	
MC_termi_LDH_1	AGTGAGCTCATCCGACGGTTTAGCTGAA TTAG	T$_{ldhL}$ from NZ7100
MC_termi_LDH_2	CCTCCATGGTGACTAGGCCTGCTGAATT TAAATGTATTAGTGCGTGTTGCGCTTTAA TGAATC	
MC_TpepN_XbaI	GCAAACAATGATCTAGACTCGAGG	pGIMCD106
MC_Pshp0064_1	ATCATTTAAATAATAAGAAAAAGCAGGACC GCCC	P$_{shp0064}$ from LMD-9
MC_Pshp0064_2	GTACCATGGGGCTTACTATTAAGAGACT AAG	
MC_FtsZ_GFP_com_1	GCTCTAGAGAATTTTCTTTAGATTCAAC CCAG	*ftsZ-gfp*$^{+}$ from pGIMCD110
MC_FtsZ_GFP_com_2	TCCCCCGGGTTATTTGTAGAGCTCATCC ATGCC	
MC_pSIP_FtsZ_GFP_1	ATAAGAATGCGGCCGCCCGCCGGCGCTT GAAGAATGG	*ftsZ* from NZ7100
MC_pSIP_FtsZ_GFP_2	ATAAGAATGCGGCCGCGAATTTTCTTTA GATTCAACCCAG	
MC_pSIP_lytA_1	CATGCCATGGGAAAGTACATACGTGAC ATTCTTTTTGG	*lytA* from NZ7100
MC_pSIP_lytA_2	CGGAATTCTTATTTTTCAAATTGTGGAT GACTCCATCCAGATGCCATGTGGATAGC AAAGCTTGG	
MC_pSIP_lytB_1	CATGCCATGGGATCACAAGCACATACA ACGGGC	*lytB* from NZ7100
MC_pSIP_lytB_2	CATGGAATTCTTATTTTTCAAATTGTGG ATGACTCCATCCAGATGCCTTAACCGTA CCTGCAAAACTTG	
MC_mutcat_lytA_1	GCTTCTGCATTCACGCAAGCTGCCTTTG C	pGIMCD117
MC_mutcat_lytA_2	GTCCATTTGTTCGTAAGGTGTGCC	
MC_pSIP_NlpC_deletion_A_1	CTAGCAGGCTCTAGAGCAGCATCTGGAT GGAGTCATCCACAATTTG	pGIMCD117 and pGIMCD118
MC_pSIP_NlpC_deletion_A_2	AAGATCTTCGCCAATGAAGGTCTTAGCA GC	
MC_pSIP_NlpC_deletion_B_2	AAGATCTTCATAAGGGATGTTGGCACTA GC	
MC_NlpC_A_1	GAAGATCTCACACCTTACGAACAAATGG ACTGC	pGIMCD117
MC_NlpC_A_2	GCTCTAGACATGTGGATAGCAAAGCTTG GTGT	
MC_NlpC_B_1	GAAGATCTCGTTTGGGGTGGTGCGAGTC TTTCG	pGIMCD118

MC_NlpC_B_2	GCTCTAGACTTAACCGTACCTGCAAAAC TTGG	
MC_pSIP_lysM_deletion_A_1	CTAGCAGGCTCTAGAGCAGTTAAAACTG GTGAAAAGGTTACG	pGIMCD117
MC_pSIP_lysM_deletion_A_2	AAGATCTTCTGTCATTGAATCGGCATTG GCAACTGC	
MC_delta_AST_LytA_1	TGCTAGAAGATCTTCCGTCTTAGTCGTA ACCTTTTCA	pGIMCD117
MC_delta_AST_LytA_2	GGCTCTAGAGCAATTGGCACACCTTACG AACAAATG	
**Validation**		
MC_pSIP_fluo_verif_1	CGCAAGAAAACTGTCAGGAAG	pGIMCD110-132
MC_pSIP_fluo_verif_2	GTCTCGGACATTCTGCTCCCG	
MC_pSIPComR	CTCAACTAACGATTTATTAGTC	pGIMCD106-107
MC_pSIPpUCOri	CGGGGTCTGACGCTCAGTGGAAC	
MC_ComRS_verif_1	CCGCCATACCACAGATGTTCC	pGIMCD101 to 102
MC_ComRS_verif_2	TCGAAACGCAGCACGATACG	

**pUC18Cm derivatives**		
**Construction**		
MC_pUC_Cm_V2_1	CCCAAGCTTCCTGCCCCGTTAGTTGAAG AAGGT	Inserts T_{ldhL}-P_{nisA}-*lytA’*, T_{ldhL}-P_{nisA}-*lytC’*, and T_{ldhL}-P_{nisA}-*mreB1’*
MC_pUC_Cm_V2_2	GGAATTCCATATGATTTTGGTTCAAAGA AGGCTAGAG	
**Validation**		
MC_pUC_verif_V2_1	GTCGATAACGCGAGCATAATAAAC	pGIMCD202, pGIMCD203, and pGIMCD206
MC_pUC_verif_V2_2	GAAGCATTTATCAGGGTTATTG	
MC_check_KOSCO_1	CAAAAATCCAAAGTAACCGC	pGIMCD208
MC_check_KOSCO_2	GCGCGTTTCGGTGATGACGGT	
MC_chromo_lytA_1	CGGTTTTGTAACTTTCGTAAC	Conditional *lytA* mutation in NZ7100
MC_chromo_lytA_2	GCACCATCCAGATTCTGACGAAC	
MC_chromo_lytC_1	GGTTTGACCAGGAAGCCAGAC	Conditional or disruptive *lytC* mutation in NZ7100
MC_chromo_lytC_2	CTACTTTAATTAAATCTAAGTC	
MC_chromo_MreB1_1	CGACAATCGCAATCACTAGTTTTC	Conditional *mreB1* mutation in NZ7100
MC_chromo_MreB1_2	GCTAGGACAGCCGTCAGTTC	
**pGIM008 derivatives**		
**Construction**		
MC_Tldh_pGIM008_1	GGAAGATCTGAAAATAAATAATCATTTC ATACG	T_{ldhL} from NZ7100
MC_Tldh_pGIM008_2	GGAAGATCTTTGCGCTTTAATGAATCGC TAAGG	
MC_pJIM_lytA_1	ACTCCATGGCCAAAGTAAATATTAAACA AGTGATGTTAGG	*lytA’* from NZ7100
MC_pGIM_lytA_PstI	AAAACTGCAGTTCGTAAGGTGTGCCAAT GAA	
MC_pGIM_lytC_PstI	AAAACTGCAGGACAATCAACACGGATTA TTGCGGCC	*lytC’* from NZ7100
MC_pGIM_lytC_SacI	CATGGAGCTCATCGAAACCATAAGCACT G	
MC_mreB1_NcoI	CATGCCATGGGATTCGGATTTGGGACAA AGAATATC	*mreB1’* from NZ7100
MC_mreB1_PvuI	AGCCCGATCGATCTCGTCCACGAATCGT TGACCC	

**Validation**		
MC_insert_pGIM008_1	GCCTTGGTTTTCTAATTTTGG	pGIMCD700, pGIMCD702, pGIMCD703, pGIMCD706
MC_insert_pGIM008_2	TGCAGGATTGTTTATGAACTC	
**pNZ8048 derivatives**		
**Construction**		
MC_GFPpAE03_PstI	AAAACTGCAGGAGCTCGAATTCGCGGCCGC	*gfp*$^{+}$ from pAE03
MC_GFPpAE03_XbaI	GCTCTAGAGGTAGCGACCGGCGCTCACATC	
MC_K7PnisGFPKpnI_3	GGGGTACCCTAGAGGTAGCGACCGGC	P_{nisA}-*gfp*$^{+}$ from pGIMCD301
MC_K7PnisGFPKpnI_5	GGGGTACCCCGATTAGTCTTATAACTATACTGAC	
**Validation**		
TR_PnisA1	TACTGACAATAGAAACATTAAC	pGIMCD301
TR_PnisA2	TATCAATCAAAGCACAC	

Supplementary Table 2. Disaccharide (Ds)-peptide composition of PG from *L. plantarum* WT and P_{nisA}-*lytA* mutant (without, N0; and with nisin, N25)

Peak[a]	Proposed structure[b,c]	% of all peaks[d]		
		WT	P_{nisA}-*lytA* (N0)	P_{nisA}-*lytA* (N25)
1	Tri missing NH_2	0.12±0.02	0.12±0.03	0.15±0.08
2	Tri	13.19±0.68	15.04±0.18	15.33±0.39
4	Tetra missing NH_2	0.93±0.03	1.07±0.20	0.76±0.18
**5**	**Di**	**1.29±0.10**	**0.30±0.04**	**0.93±0.08**
7	Tetra	5.74±0.16	5.48±0.07	6.69±0.49
**8**	**Tri-D-ALa-mDAP(NH_2)**	**1.19±0.07**	**0.23±0.03**	**0.82±0.07**
9	Tri-D-Ala-mDAP(NH_2)-D-iGln-L-Ala	0.27±0.04	0.13±0.04	0.29±0.07
10	Tri (OAc-M)	0.81±0.11	0.52±0.03	0.38±0.13
12	Tri (OAc-M)	5.95±0.54	4.06±0.15	3.24±0.57
14	Tri (OAc-G)	1.65±0.12	0.80±0.11	1.07±0.12
**15**	**Di (Ac)**	**0.54±0.05**	**0.07±0.01**	**0.20±0.08**
16	Tetra-D-Ala-mDAP (NH_2)-D-iGln-L-Ala	0.13±0.004	0.07±0.004	0.09±0.003
	Tetra (Ac)	0.13±0.004	0.07±0.004	0.09±0.003
17	Tetra (Ac)	1.74±0.11	0.86±0.02	0.97±0.09
**18**	**Tri-D-ALa-mDAP(NH_2) (Ac)**	**0.57±0.08**	**0.06±0.01**	**0.20±0.07**
19	Tri-D-Ala-mDAP(NH_2)-D-iGln-L-Ala (Ac)	0.22±0.01	0.27±0.02	0.30±0.02
	Tetra (Ac)	0.22±0.01	0.27±0.02	0.30±0.02
22	Tetra-D-Ala-mDAP(NH_2)-D-iGln-L-Ala-missing NH2	0.88±0.18	0.61±0.03	0.44±0.08
23	Tri (2Ac)	0.82±0.08	1.09±0.07	1.06±0.47
	Ds-peptides	36.39±2.37	31.14±1.07	33.31±3.02
	Ds-peptides resulting from D-iGln-mDAP(NH_2) cleavage	3.59±0.29	0.66±0.08	2.16±0.29
	% D-iGln-mDAP(NH_2) cleaved Ds-peptides/total Ds-peptides	9.87	2.12	6.48

[a] Peak numbers and structures were previously assigned for *L. plantarum* NZ7100 (Bernard et al., 2011) and are displayed on Figure S1. The retention time of peak n°5 containing disaccharide-dipeptide (Di) was validated using a purified Di previously characterized by mass spectrometry.
[b]Di, disaccharide dipeptide (L-Ala-D-iGln); Tri, disaccharide tripeptide (L-Ala-D-iGln-mDAP(NH_2)); Tetra, disaccharide tetrapeptide (L-Ala-D-iGln-mDAP(NH_2)-D-Ala); Disaccharide, GlcNAc-MurNAc; (NH_2), amidation; Ac, acetylation on MurNAc or GlcNAc; OAc-M, O-acetylMurNAc; OAc-G, O-acetylGlcNAc.
[c]Muropeptides resulting from cleavage of a D-iGln-mDAP(NH_2) bond are indicated in bold.
[d]Percentage of each peak was calculated as the ratio of the peak area over the sum of areas of all the peaks identified on the corresponding chromatogram. Mean values of three independent extractions ± standard deviations.

Reference

Bernard, E., Rolain, T., Courtin, P., Guillot, A., Langella, P., Hols, P. et al. (2011a). Characterization of O-acetylation of N-acetylglucosamine: a novel structural variation of bacterial peptidoglycan. J. Biol. Chem. 286, 23950-23958. 10.1074/jbc.M111.241414 [doi].
